# Optical, Electrochemical, Third-Order Nonlinear Optical Investigations of 3,4,5-Trimethoxy Phenyl Substituted Non-Aqueous Phthalocyanines

**DOI:** 10.3389/fchem.2021.713939

**Published:** 2021-09-09

**Authors:** K. S. Srivishnu, Dipanjan Banerjee, Ramya Athira Ramnagar, Jagannath Rathod, Lingamallu Giribabu, Venugopal Rao Soma

**Affiliations:** ^1^ Polymers and Functional Materials Division, CSIR-Indian Institute of Chemical Technology, Hyderabad, India; ^2^ Academy of Scientific and Innovative Research (AcSIR), Ghaziabad, India; ^3^ Advanced Centre of Research in High Energy Materials (ACRHEM), University of Hyderabad, Hyderabad, India

**Keywords:** phthalocyanine, Z-scan, two-photo absorption, femtosecond, optical switching

## Abstract

A new series of non-aqueous phthalocyanines having 3,4,5-trimethoxy phenyl group at peripheral positions in which the central cavity possessing Cu(II), Zn(II), and without metals has been synthesized, and its absorption, fluorescence (steady-state and excited state lifetimes), electrochemical, and third-order nonlinear optical (NLO) properties were evaluated. Absorption studies data suggest that all three phthalocyanines obey Beer–Lambert’s law, and the redox properties indicate that both oxidation and reduction reactions are macrocyclic centered. The singlet quantum yields were measured in different solvents and were found to be in the range of 0.2–0.5 in the case of free-base, whereas it was in the range of 0.1–0.5 in zinc derivative, while the time-resolved fluorescence data revealed lifetimes of typically a few ns. The third-order NLO properties were investigated using the Z-scan technique with kilohertz (for retrieving true electronic nonlinearities) and megahertz repetition rate femtosecond pulses at 800 nm. Intensity-dependent Z-scan studies revealed robust NLO coefficients for solutions and thin films (two-photon absorption cross-sections of 9,300–57,000 GM) of these molecules suggesting a strong potential for optical switching, imaging, and optical limiting applications.

## Introduction

In the current era of optoelectronics, nonlinear optics (NLO) and related technologies play a significant role in its evolution (Dini et al., 2016; Gounden et al., 2020; Mrinalini et al., 2021). To revive the study of the potential of NLO in the field of telecommunication, photonics, biomedicine, and optical signal processing, several NLO moieties were designed through various π-conjugated organic molecules (Mardar, 2006; Bures et al., 2012; Sofiani et al., 2016; Biswas et al., 2020). For this reason, a great variety of small organic molecules and macrocyclic systems, such as porphyrins (Rao et al., 2000; Senge et al., 2007; Swain et al., 2014a; Katturi et al., 2020b; Rathi et al., 2020; Vijisha et al., 2020; Kumar et al., 2021a), phthalocyanines (de la Torre et al., 2004; Kumar et al., 2007; Swain et al., 2014b; Zhang et al., 2014; Bharati et al., 2018; Venkatram et al., 2018a; Venkatram et al., 2018b; Bhattacharya et al., 2019a; Bhattacharya et al., 2019b; Bhattacharya et al., 2021), corroles (Katturi et al., 2020a; Lu et al., 2021), porphycenes (Sarma et al., 2011; Swain et al., 2012; Pati et al., 2020), and BODIPY dyes (Ngoy et al., 2019), have been investigated for their NLO properties. Of these systems, we are particularly interested in phthalocyanine-based macrocyclic systems toward these applications. Phthalocyanines (Pcs) are large aromatic pigment molecules composed of four isoindole units linked by nitrogen atoms (Giribabu et al., 2008). Unlike porphyrins, phthalocyanines are human-made compounds which have a wide range of advantages in diversified areas such as sensor technology, catalysts, electrochromism, liquid crystals, photodynamic therapy, and energy storage devices to name a few (Gregory, 2000; Zagal et al., 2010; Tuncel et al., 2012; Kuzmina et al., 2019; Buyukeksi et al., 2020; Kumar et al., 2021b; Halaskova et al., 2021). Because of their electronic delocalization, innumerable properties arise which make them of great potential in various fields of science and technology. Despite all these applications, phthalocyanines are aggregates even at micromolar concentration due to planarity. In addition to this, the phthalocyanine macrocycle is insoluble in almost all organic solvents and is a major hurdle for many optoelectronic applications. To overcome these constraints, one has to incorporate alkyl or alkoxy chains either at the peripheral or non-peripheral positions of phthalocyanines (Singh et al., 2014). Furthermore, phthalocyanines offer for hosting more than 70 different elements in the central cavity. Phthalocyanines possess an absorption band in the vicinity of 350 nm (Soret band) and one or two bands in the 500–700 nm region (Q band/s) with high molar absorption coefficient (ε = 10^5^) with high triplet state quantum yields and long lifetimes. Furthermore, the phthalocyanine macrocycle has rich electrochemistry, and it involves both oxidation and reduction reactions. The incorporation of alkyl or alkoxy groups as well as metals in its central cavity not only affects its optical but also its redox reactions.

**FIGURE 1:**
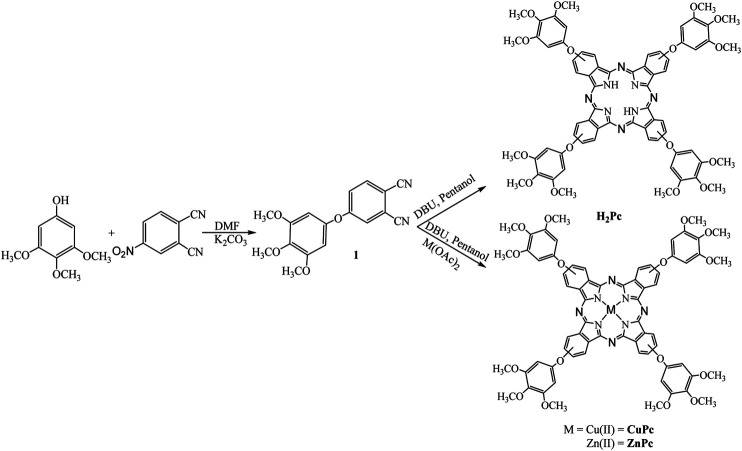
Synthetic scheme of phthalocyanines.

Recent research trends have undeniably demonstrated the need for the development of optical limiters and nonlinear materials for the protection of sensitive optical devices and components from laser-induced damage (Dini et al., 2016). The study of optical nonlinearities, i.e., intense light when interacting with matter, is significant for the development of various photonic/optoelectronic devices. Generally, electronic distribution of the material does not get disturbed when light with low intensity falls on it, whereas when light with high intensity falls on the matter, it has enough power to pass the threshold limit which leads to the change in properties of the transmitted light. Optical nonlinearity arises at the molecular level due to the presence of delocalized π electrons. These electrons are easily polarized by the strong electric field of the input laser pulses. Phthalocyanines and metallated phthalocyanines (MPc) do have all the structural requirements for high polarizable π-systems and demonstrate large nonlinear optical (refraction/absorption) coefficients with ultrafast response times (typically in the sub-picosecond and femtosecond time scales). Along with these, Pcs also exhibit strong optical limiting mechanism which is observed due to phenomena such as reverse saturation absorption (RSA) and two-photon absorption (TPA) processes. Due to intersystem crossing (from S_1_ to T_1_ states) phthalocyanines exhibit strong RSA when excited with long (nanosecond) laser pulses. In this study, we designed, synthesized, and characterized a series of phthalocyanines possessing the 3,4,5-trimethoxy phenol group at their peripheral positions and Cu(II) or Zn(II) metals at their central cavities (see Figure 1 schematic depicting the synthesis procedure). The detailed optical, electrochemical, and NLO properties of these phthalocyanines are thoroughly studied. The NLO properties were evaluated in solution and thin film forms. The input wavelength was 800 nm and femtosecond (kHz and MHz repetition rates) laser pulses were used for the NLO studies. Complete NLO coefficients such as nonlinear refraction (n_2_), nonlinear absorption/two-photon absorption coefficients (β), third-order nonlinear optical susceptibility [χ^(3)^], and two-photon absorption cross-sections [σ2 or σT_PA_] were extracted from the Z-scan data.

## Experimental Details

Materials: 4-Nitrophthalonitrile, 3,4,5-trimethoxyphenol, 1-pentanol, and 1,8-diazabicyclo[5.4.0] undec-7-ene (DBU) were procured from Sigma Aldrich and used without any further purification. Copper acetate, zinc acetate, and potassium carbonate were purchased from Finar Ltd. and used as such. All the solvents, dichloromethane (DCM), tetrahydrofuran (THF), dimethylformamide (DMF), toluene, n-hexane, acetonitrile, and methanol, were purchased from SD Fine Chemicals Ltd. and were dried before use.

### Synthesis

***3,4,5-Trimethoxyphenoxy phthalonitrile (1):*** 4-Nitrophthalonitrile (2 g, 11.56 mmol) and 3,4,5-trimethoxyphenol (1.57 g, 11.56 mmol) were taken in a clean dry round bottom flask. To this, potassium carbonate (8.32 g, 22.2 mmol) and dimethylformamide (25 ml) were added and stirred for 24 h at 60°C under N_2_ atmosphere. The reaction mixture was poured onto ice and the obtained product was filtered off. The obtained solid was further purified by silica gel column chromatography by eluting with hexane-ethyl acetate (85:15 v/v) to get the desired compound. Anal. calcd for C_17_H_14_N_2_O_4_%(310.10): C, 65.80; H, 4.55; N, 9.03. Found C, 65.84; H, 4.52; N, 9.01. ^1^H NMR (300 MHz, CDCl_3_): 7.75 (d, 2H), 8.20–8.40 (m, 3H), 3.90 (s, 3H), 3.85 (s, 6H).

***Synthesis of TmPc***: 3,4,5-Trimethoxyphenoxy phthalonitrile (0.20 g, 0.645 mmol) was taken in a clean dry 25 ml round bottom flask in which 1-pentanol was added and stirred at 200°C. After 15 min, DBU was added in catalytic amount and continued to reflux for 8 h under nitrogen atmosphere. The reaction was cooled down to room temperature and precipitated with methanol, and washed with hexane. It was further purified with silica gel column chromatography using dichloromethane. The obtained product was dried and the yield was 40%. Anal. calcd for C_72_H_62_N_4_O_16_%(1,239.30): C, 69.78; H, 5.04; N, 4.52. Found C, 69.80; H, 5.02; N, 5.51. MALDI-TOF MS calcd m/z (C_72_H_62_N_4_O_16_) 1,239.30, found 1,241.41.

***Synthesis of ZnTmPc***: 3,4,5-Trimethoxyphenoxy phthalonitrile (0.2 g, 0.645 mmol) and zinc acetate (0.20 g, 1.098 mmol) were taken in a clean dry 25 ml round bottom flask in which 1-pentanol was added and stirred at 200°C. After 15 min, DBU was added in catalytic amount and continued to reflux for 6 h under nitrogen atmosphere. The reaction was cooled down to room temperature and precipitated with methanol, and washed with hexane. It was further purified with silica gel column chromatography using dichloromethane. The obtained product was dried and the yield was 45%. Anal. calcd for C_72_H_60_N_4_O_16_Zn %(1,302.66): C, 66.39; H, 4.64; N, 4.30. Found C, 66.40; H, 4.02; N, 4.31. MALDI-TOF MS calcd m/z (C_72_H_60_N_4_O_16_Zn) 1,302.66, found 1,304.49.

***Synthesis of CuTmPc*:** Similar procedure was followed as of Zn-TmPc; copper acetate was used instead of zinc acetate, and the yield was about 35%. Anal. calcd for C_72_H_60_N_4_O_16_Cu %(1,300.83): C, 66.48; H, 4.65; N, 4.31. Found C, 66.46; H, 4.62; N, 4.31. MALDI-TOF MS calcd m/z (C_72_H_60_N_4_O_16_Cu) 1,300.83, found 1,304.49.

***Thin films:*** The thin films were prepared by coating 2 mM concentration solution of Pc dissolved in DCM on a glass plate. Glass plates of size 2.5 × 2.5 mm each were cut and washed with water and acetone under sonication and dried at 100°C. A dried glass plate was inserted on the base of the spin coater and 0.1 ml of solution was added using a micropipette onto the glass plate to obtain the thin film at a speed of 2000 rpm. Later, the glass plate was dried in a hot furnace at 60°C to obtain the thin films. The thickness was monitored using a profilometer, and the obtained values were in the range of 47–56 µm. See Supplementary Figure S18 for thickness data and Supplementary Figure S19 for absorption data of the thin films. It was observed that the Q and B bands of the absorption spectra in thin films were broadened and slightly red-shifted.

### Nonlinear Optical Studies

Z-scan is a promising NLO technique found by Sheik Bahae and co-workers (1990). We have carried out the Z-scan experiments to probe the NLO properties of the title molecules extracting clear evidence about the sign and magnitude of NLO susceptibility [χ^(3)^] in an ultrafast excitation regime. Now, the input excitation beam being a Gaussian profile, the normalized transmittance can be mathematically followed up properly by bringing in the multi-photon absorption theory. In an open and closed aperture configuration, the normalized transmittance turns out to be attending the mathematical expressions as per the following equations. The generic multi-photon absorption-influenced transmittance nature (Kumar et al., 2007; Anusha et al., 2012; Bhattacharya et al., 2021) of the Z-scan process can be signified in a mathematical form as follows:

$$Transmittance\;T_{OA\left(nPA\right)}=\frac{1}{{\left[1+\left(n-1\right)\alpha_{n}L{\left(I_{0}/\left(1+{\left(\frac{z}{z_{0}}\right)}^{2}\right)\right)}^{n-1}\right]}^{\frac{1}{n-1}}}$$ [1]

While in the case of TPA phenomena, it can be reduced to a simpler form as

$$Transmittance,T_{OA\left(2PA\right)}=\frac{1}{1+\beta L_{eff}\left(\frac{I_{00}}{1+{\left(\frac{z}{z_{0}}\right)}^{2}}\right)}$$ [2]

And in the case of the closed aperture, it is supposed to be

$$Transmittance,T_{CA\left(2PA\right)}=\left(1\pm \left(\frac{4\left(\frac{z}{z_{0}}\right)\Delta \Phi }{\left[9+{\left(\frac{z}{z_{0}}\right)}^{2}\right]\left[1+{\left(\frac{z}{z_{0}}\right)}^{2}\right]}\right)\right)$$ [3]

So, here the sample effective is estimated by ${\text{L}}_{\text{eff}}\left({\text{cm}}^{-1}\right)=1-{\text{e}}^{-\text{αL}}/\text{α}\left(\text{TPA}\right)$, Beam waist at focal point (z=0), ${\text{ω}}_{0}\left(\text{mm}\right)=2\text{f}.\text{λ}/\text{π}.\text{d}$, the measured Rayleigh Range would be ${\text{z}}_{0}\left(\text{mm}\right)={\text{πω}}_{0}^{2}/\text{λ}$, λ is the wavelength and ‘d’ is the incident beam waist at focal point. Now, the second order Non-linear refractive index is subjectively related to the $\text{ΔΦ},\;\text{as}\;{\text{n}}_{2}\left({\text{cm}}^{2}/\text{W}\right)=\text{ΔΦ}/{\text{I}}_{0.}{\text{L}}_{\text{eff}}.\text{k}=\text{ΔΦ}.\text{λ}/{\text{I}}_{0.}{\text{L}}_{\text{eff}}.2\text{π}$. Now, over and above we are able to estimate both real, imaginary ${\text{χ}}^{\left(3\right)}$ from the relations as follows, $\text{Im }\left|{\text{χ}}^{\left(3\right)}\right|\left({\text{m}}^{2}/{\text{V}}^{2}\right)=\text{c}\epsilon_{0}{\text{λn}}_{0}^{2}{\text{α}}_{2}\left(\text{m}/\text{W}\right)/2\text{π}$, $\text{Re}\left|{\text{χ}}^{\left(3\right)}\right|\left({\text{m}}^{2}/{\text{V}}^{2}\right)=2\text{c}\epsilon_{0}{\text{n}}_{0}^{2}{\text{n}}_{2\;}\left({\text{m}}^{2}/\text{W}\right)$ and finally ${\text{χ}}^{\left(3\right)}$ total will emerge from the above both.Initially, the NLO properties have been investigated in solution form utilizing both MHz and kHz femtosecond pulses and, subsequently, the same molecules have been studied in the form of thin films. For fs MHz studies, we had engaged 80 MHz repetition rate, 150 fs laser pulses at 800 nm and we performed thorough intensity dependent nonlinear absorption studies in the open aperture configuration. In the MHz Z-scan setup, a 10 cm lens was installed to focus the input beam, along with a photo-sensor and power meter, interfaced using a LabVIEW controlled PC. Whereas in case of femtosecond amplifier kHz (1 kHz, 50 fs, 800 nm), a lock-in amplifier has been utilized with the silicon photo detector. Here also we performed intensity dependent Z-scan studies. In both scenarios, an iris has been installed to vary the collection window of the collecting plano-convex lens as well as primarily to study the closed aperture configuration. A quartz cuvette has been utilized to store the sample to interact with the ultrafast pulses. Solutions of different compounds (TMPc, Cu-TMPc,Zn-TMPc) have been prepared maintaining a similar concentration of ∼1mM [solvent being dichloromethane (DCM)] for the NLO studies to give clear results. Significant NLO coefficients have been extracted out through rigorous analysis (theoretical fittings) to come to a conclusive decision about the application of these novel phthalocyanines.

## Methods and Instrumentation

### Characterization

All the ^1^H NMR spectra were recorded in CDCl_3_ solution using a spectrophotometer (AVANCE, 300 MHz). Matrix-assisted laser desorption ionization time-of-flight (MALDI–TOF) mass spectrometry measurements were performed on Shimadzu Biotech AximaPerformance 2.9.3.20110624: Mode was Reflectron-Hi Res, Power: 85 using TMS as standard. FT-IR spectra (with KBr pellets) were recorded on a Bruker spectrometer.

### Optical Studies

A UV-Visible-NIR spectrophotometer (Shimadzu UV-3600) was used to record the absorption spectra of the title compounds. A spectrofluorometer (Fluorolog-3, Spex model, JobinYvon) was utilized to obtain the steady-state fluorescence spectra of solutions. The optical density at the excitation wavelength (λex) was ∼0.06. Zinc tert-butyl phthalocyanine (=0.37 in benzene) was used as a reference to calculate the fluorescence quantum yields (ϕ) by integrating their fluorescence bands (Lawrence and Whitten, 1996). Picosecond time-correlated single photon counting setup (consisting of FluoroLog3-Triple Illuminator, IBH Horiba JobinYvon) was employed to record the fluorescence lifetime data using a picosecond light emitting diode laser (NanoLED, λexof670 nm) as the source of excitation. The decay curves were obtained by from the fluorescence emission maxima of the phthalocyanine (λem = 700 nm). A photomultiplier tube (R928P from Hamamatsu) was used as the detector. The lamp profile was achieved by placing a scattered (dilute Ludox solution in water) in place of the sample. The width of the instrument function was limited by FWHM of the excitation source, which was ∼635 ps at 670 nm. The obtained decay curves were analyzed using nonlinear least-squares iteration procedure using a software [IBH DAS6 (version 2.3) decay analysis]. The quality of the fits was adjudicated by the χ^2^ values and the residual distribution.

### Electrochemical Studies

Electrochemical measurements were performed on a potentiostat that was computer controlled [CH instruments, model CHI 620C]. The experiments were performed in solutions of respective phthalocyanines in DCM (using 1 mM concentration) and at a scan rate of 100 mV/s using 0.1 M tetrabutyl ammonium perchlorate as the supporting electrolyte. The working electrode was glassy carbon, and a standard calomel electrode was the reference electrode while a platinum wire was used as an auxiliary electrode. After cyclic voltammograms (CVs) have been recorded, ferrocene was added, and a second voltammogram was measured.

#### Theoretical Calculations

By using the Gaussian 09 package, loaded on a personal computer, all the theoretical analyses were performed (following Becke, 1993). The optimized energy minimized structures of all three phthalocyanines were found to be stable with global minimal energy. These studies were accomplished using the density functional theory (DFT) at B3LYP hybrid functional theory at 6-31G(d,p) basis set in the Gaussian program (see Peterson and Al-Laham, 1991; Frisch et al., 2009). The excited state properties such as 1) percentage of molecular contribution, 2) oscillatory strength, and 3) singlet transition energy in the tetrahydrofuran solvent were obtained by performing the time-dependent DFT (TD-DFT) calculations. The integral equation formalism polarizable continuum model within the self-consistent reaction field theory was used in the TD-DFT calculations to describe with M06-2x function and the solvation of the phthalocyanines in THF (Miertus et al., 1981; Cossi et al., 1996). GaussSum 2.2.5 was employed to simulate the most important portions of the absorption spectra and to construe the nature of various transitions (O’Boyle et al., 2008; Dennington et al., 2009).

## Results and Discussions

The synthetic approach utilized for preparing three phthalocyanines is displayed in Figure 1. The compound 4-(3,4,5-trimethoxyphenoxy)phthalonitrile (**1**) was synthesized by the condensation of 3,4,5-trimethoxyphenol with 4-hydroxy phthalonitrile using K_2_CO_3_ in DMF. The cyclo tetramerization of **1** was achieved using DMF and pentanol method to get the free-base phthalocyanine (**TmPc**) and the corresponding metallo derivatives were achieved by using DBU, pentanol with respective metal acetates. All three phthalocyanines are characterized by various spectroscopic and electrochemical methods. The results obtained from the elemental analysis of all three compounds were found to be satisfactory. The presence of molecular ion peak in MALDI-Ms spectra of each compound confirmed the respective phthalocyanine (see Supplementary Figures S1–S8 for all the details).

### Optical and Electrochemical Properties

**FIGURE 2:**
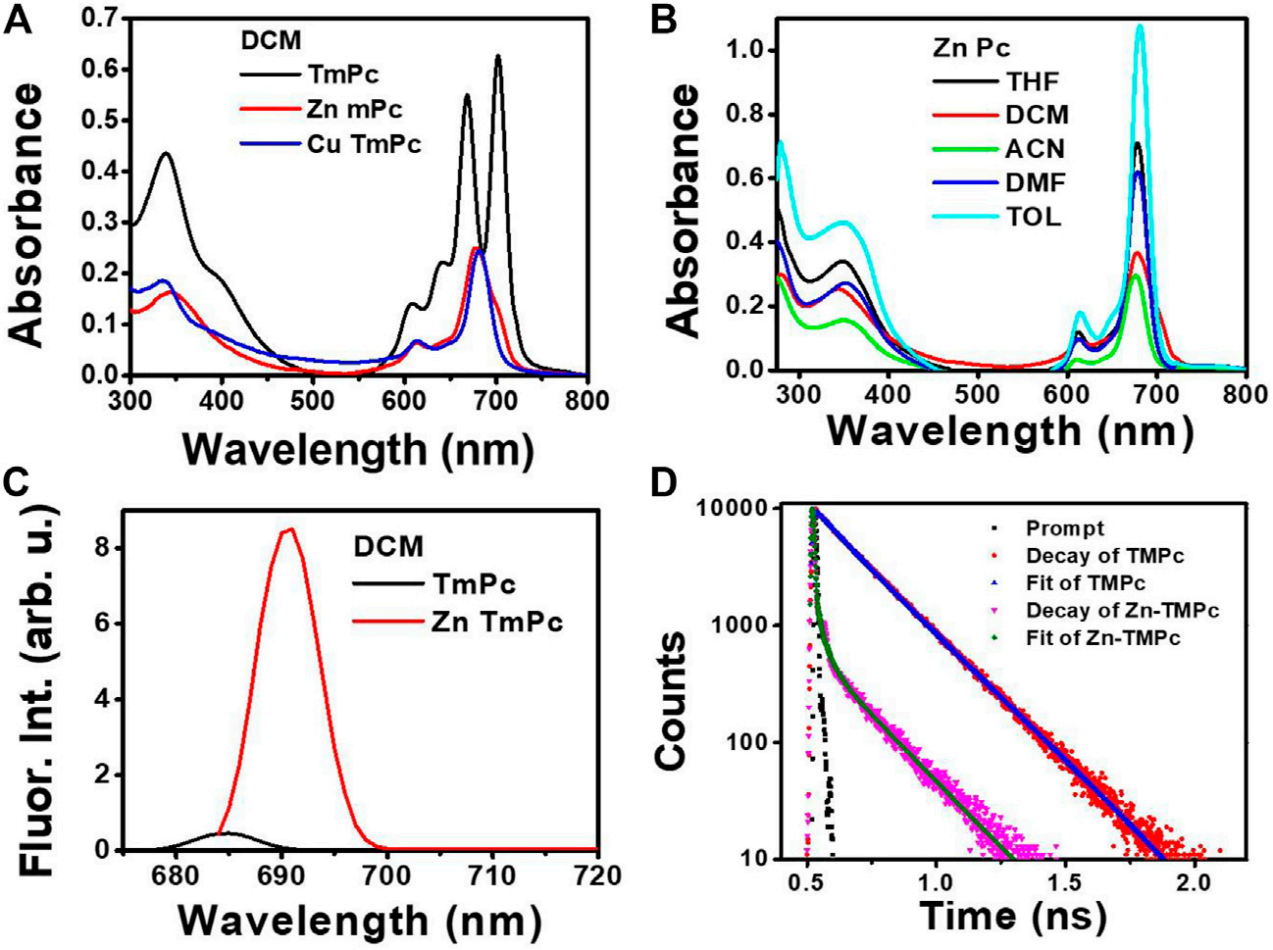
**(A)** Absorption spectra of TmPC, CuTmPc, and ZnTmPc in the DCM solvent **(B)** Absorption spectra of ZnTmPc in different solvents **(C)** Emission spectra of TmPc and ZnTmPc in the DCM solvent **(D)** Fluorescence decay curves of TmPc and ZnTmPc in the DCM solvent. “arb. u.” stands for arbitrary units.

It is well known in literature that phthalocyanine macrocycles are usually insoluble in organic solvents mainly due to the planarity of the macrocycle that promotes aggregation even at very low (micromolar) concentrations (Giribabu et al., 2008). This can be minimized by incorporation of various organic substituents at its peripheral position and metals at its central cavity (Singh et al., 2014). The optical absorption spectra of all three compounds, i.e., **TmPc**, **CuTmPc**, and **ZnTmPc** were measured in the DCM solvent and the respective absorption spectra are displayed in Figure 2A. The corresponding absorption maxima (λ_max_) and logarithmic value of the molar extinction coefficients (log ε) are presented in Table 1. The less intense peak in the 300-400 nm spectral region is attributed to the Soret band, which arises because of the transition from deeper π-levels to LUMO. In contrast, the intense peak(s) in 600-750 nm region belongs to the Q band, which arises due to well-known π-π* transitions. The lower intensity at higher energy side of Q band, i.e., 625 nm arises due to the aggregation of phthalocyanine. Metallophthalocyanines typically exhibit a single Q band in the visible spectral region whereas in the corresponding free base Pc, a split Q band is noticed which is due to lowering of symmetry, which is found to lie in 640-690 nm region. In addition, we have also measured absorption spectra of all three compounds in non-coordinating solvents such as toluene and acetonitrile, and coordinating solvents such as THF and DMF so that the solvent coordinates at axial positions of **CuTmPc** and **ZnTmPc** that lead to further minimization of aggregation [Figure 2B and Supplementary Figures S9–S11]. Furthermore, we measured emission spectra of both **TmPc** and **ZnTmPc** by exciting at 650 nm in various solvents, namely, toluene, DCM, acetonitrile, THF, and DMF [Figure 2C and Supplementary Figure S12]. It is well known and reported in literature that copper is a paramagnetic metal and due to heavy atom effect the emission is almost quenched and will be not be observed in the spectra. The corresponding emission maxima and quantum yields are presented in Table 1. The quantum yield data presented in Table 1 evaluates the polarity of the solvents. It was observed that the quantum yields are reduced when the polarity of the solvent increased due to aggregation phenomena and it influences even in the excited state life time. The radiative decay life times of newly synthesized phthalocyanines in different solvents (toluene, DCM, THF, DMF, and acetonitrile) are demonstrated in Figure 1D and Supplementary Figure S13. The corresponding excited state (singlet) lifetime data are summarized in Table 1.

**TABLE 1:** Optical and electrochemical data of the three phthalocyanines investigated.

Compound	λ_max_, nm (log ε)^a^ M^−1^ cm^−1^	λ_em, max_ nm (φ_f_)^b^	τ_f_, ns (A%)^c^	E_0-0_ (eV)^d^	E_1/2_ vs. SCE^e^	
					Ox	Red
**TmPc**	693 (4.79)	698 (0.47)	5.33 (100)	1.78	0.85, 1.26	–1.00
	659 (4.64)	—	—	—	—	—
	642 (3.98)	—	—	—	—	—
	618 (3.75)	—	—	—	—	—
	345 (4.25)	—	—	—	—	—
**CuTmPc**	680 (4.38)	–	–	1.80	0.70, 1.27	–1.20
	618 (3.25)	—	—	—	—	—
	340 (4.12)	—	—	—	—	—
**ZnTmPc**	677 (4.40)	689 (0.15)	0.99 (11)	1.82	0.88, 1.28	–0.98
	620 (3.56)	—	0.09 (54)	—	—	—
	350 (4.09)	—	5.33 (35)	—	—	—

a Error limits: λ_max_, ± 1 nm, log ε, ± 10%.

b Error limits: λ_em_, ± 1 nm, φ_f_ ± 10%.

c Singlet excited lifetime measured in the DCM solvent.

d E_0–0_ was determined from the intersection of normalized absorption and emission spectra.

e The oxidation potentials of the dyes were measured in THF with 0.1 M tetrabutylammoniumhexafluorophosphate (TBAPF6) with a scan rate of 100 mV s^−1^ (vs. SCE).

The redox behavior of all three phthalocyanines was measured by using cyclic voltammetric technique (CV). In general, phthalocyanines tend to lose one or two electrons in its oxidation state while it gains one to four electrons in its reduction state. To appraise the redox potentials of all three phthalocyanines, we performed CV using tetrabutylammonium hexfluorophosphte (TBAPF_6_) in DCM as supporting electrolyte and ferrocene as external standard. Supplementary Figure S13 illustrates CV of all three phthalocyanines and the corresponding redox data presented in Table 1. The redox reactions are either cathodically or anodically shifted while metallation. All three phthalocyanines exhibited two sequential oxidation potentials at 0.85 and 1.26 V vs. SCE (**TmPc**), 0.70 and 1.27 V vs. SCE (**CuTmPc**), while **ZnTmPC** at 0.88 and 1.28 V vs. SCE. In addition, one quasi-reversible reduction was observed in all three macrocycles. Based on literature information, all redox behaviors are macrocyclic centered and certainly not metal centered.

To gain further insights into the structural, optical, and redox properties of these phthalocyanines, we have adopted DFT and TD-DFT techniques [with a functional basis set of the B3LYP/6-31G (d,p) level] from the Gaussian 09 package. The optimized structures of all three phthalocyanines obtained are displayed in Supplementary Figure S14. The optimized structures suggested that the phthalocyanine macrocycle is in a plane where the substituent 3,4,5-trimethoxyphenyl moiety is in tilted position and this tends to minimize the aggregation of the phthalocyanine, which is reflected in the absorption spectra [see Figure 2A].

### Quantum Mechanical Calculations

**FIGURE 3:**
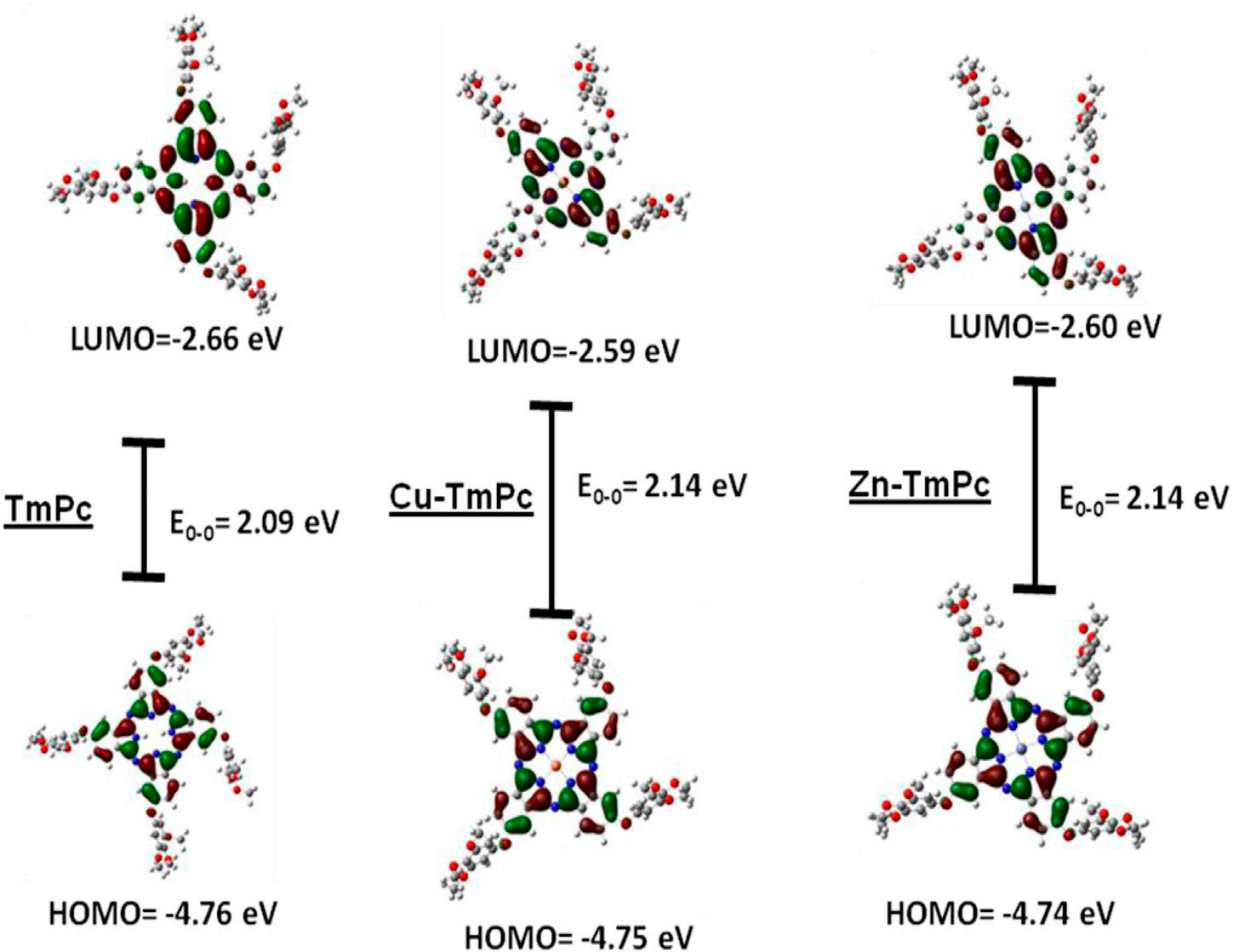
Frontier HOMO and LUMO of **TmPc**, **Cu-TmPc** and **Zn-TmPc**.

Figure 3 and Supplementary Table S1 depict the HOMO, LUMO, and the HOMO-LUMO gap energies and ground state dipole moment (in Debye units), respectively. The energy levels and electron density distribution of all three phthalocyanines have shown same results estimated at HOMO: 4.75 eV and LUMO 2.65 eV with energy band gap of 2.10 eV (Figure 2). Furthermore, HOMO to HOMO-2 and LUMO+2 to LUMO energy distributions and their levels are shown in Supplementary Figures S15–S17. These data suggest that HOMOs are occupied by higher electron density on the trimethoxyphenyl ring while LUMOs are occupied by more electron clouds on the acceptor Pc macrocycle. In general, the metallation of phthalocyanine central cavity reduces the HOMO-LUMO gap. However, in the present case, the HOMO-LUMO gap was found to be marginally enhanced, possibly due to the presence of electron releasing groups at its peripheral positions (Louazri et al., 2015; Seifallah et al., 2020).

### Nonlinear Optical Measurements

Promising novel organic molecules, possessing significant nonlinear optical (NLO) characteristics, are in high demand for various device applications in the fields of photonics, biomedical imaging, optical communications, etc., (Anusha et al., 2015; Venkatram et al., 2008a). All these organic molecules, like phthalocyanines (Rao et al., 2000; Maya et al., 2003; Swain et al., 2014b; Bharati et al., 2018; Venkatram et al., 2008b), porphyrins, and corroles (Anusha et al., 2012; Sarma et al., 2011) are reported frequently possessing strong third-order NLO coefficients and cross-sections (Swain et al., 2014a; Bhattacharya et al., 2019a; Clavian et al., 2019; Katturi et al., 2020a; Katturi et al., 2020b; Pati et al., 2020; Rathi et al., 2020; Kumar et al., 2021). Phthalocyanines generally have strong colors and are used as dyes. Phthalocyanines, being categorically from the aromatic organic macromolecules group, possess a huge number of delocalized electrons. The central cavity ring component in the structure of these phthalocyanines play a crucial role in their response toward laser-matter interaction. In a recent work, Cu, Zn incorporated tri-methoxy-phenoxy-phthalocyanine (**TMPc**, **Cu-TMPc**, **Zn-TMPc**) were synthesized in the form of macromolecules utilizing the matrix-assisted laser desorption ionization (MALDI) technique and extensive nonlinear optical measurements were carried out using the same.

To begin with, solution forms of these novel phthalocyanines were probed with MHz femtosecond pulses (at 800 nm) and the light-matter nonlinear interaction (Sutherland et al., 2003) were studied thoroughly. We have obtained the Z-scan experimental results and were able to extract various NLO parameters such as 1) two photon absorption coefficient β (cm.W^−1^), 2) two-photon absorption cross section σ_TPA_ (GM), 3) nonlinear refractive index n_2_ (cm^2^.W^−1^), and 4) nonlinear optical susceptibility index [χ^(3)^] by fitting the experimental data to mathematical/theoretical expressions**.** All the phthalocyanine molecules have exhibited purely two-photon absorbed reverse saturation absorption (RSA) nature in open aperture configuration while interacting with 80 MHz, 150 fs pulses at 800 nm. Intensity-dependent study has been carried out for all the samples, which clearly revealed an increase in two-photon absorption coefficient (β) with an increase in intensity order gradually. The RSA nature of the open aperture curves signifies the clear downfall in output transmittance, after the sample, with fine increase in intensity while approaching the focal point, clarifying the occurrence of nonlinear absorption. Whereas, in closed aperture mode, prominent peak-valley nature has been observed for all three phthalocyanines, which stands for a defocusing Kerr lens effect taking place due to high intensity dependence.

**FIGURE 4:**
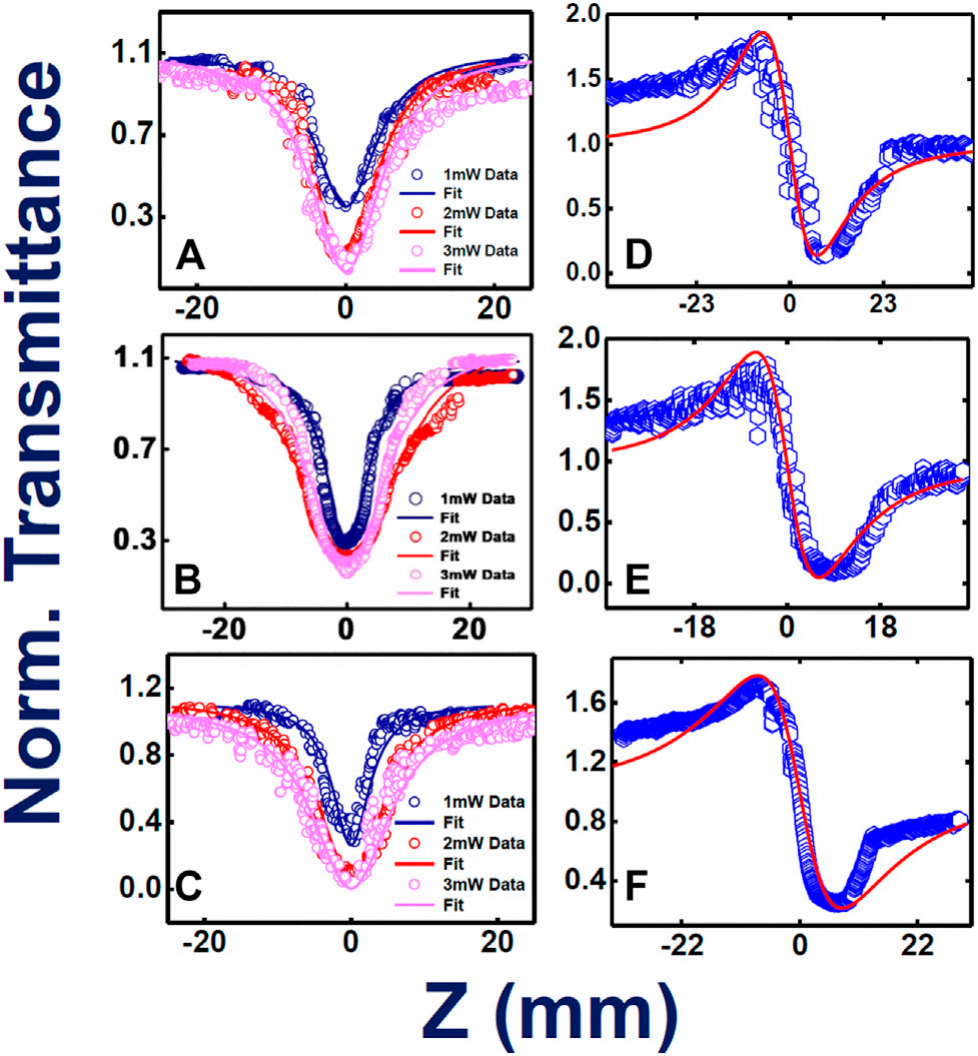
Femtosecond MHz pulses experimentally and theoretically fitted Z-scan data (intensity dependent) open aperture **(A–C)** and closed aperture configurations **(D–F)** for **TMPc**, **Cu-TMPc**, **Zn-TMPc**, respectively, in solution form. Open symbols are the experimental data while the solid lines represent theoretical fits.

Figures 4A–C illustrate the intensity varied open aperture mode Z-scan data obtained for the phthalocyanines **TMPc**, **Cu-TMPc**, **Zn-TMPc**. The peak intensity has been varied from 8 MW/cm^2^ to 16 MW/cm^2^ and finally up to 24 MW/cm^2^, and we measured the corresponding normalized transmittance with the scanning position on and around the Rayleigh range. The estimated two–photon absorption coefficient (β) values for **TMPc** have been found increasing from (3.8–42) × 10^−6^ cm.W^−1^. Similarly, the β values corresponding to **Cu-TMPc** molecules have been found escalating from (4-11) × 10^−6^ cm.W^−1^, and the same for **Zn-TMPc** phthalocyanine has been observed to be in the range of (4.2–140) × 10^−6^ cm.W^−1^. **Zn-TMPc** has clearly shown significantly superior nonlinearity, making it a potential candidate for photonic applications. The two-photon absorption cross-sections (σ_2_) have also been calculated to be found in the order of ∼10^8^ GM. From Figures 4D–F, closed aperture Z-scan data have revealed the nonlinear refractive index (n_2_) values as of 3.6 × 10^−11^, 3.5 × 10^−11^, 5.7 × 10^−11^ cm^2^/W for **TMPc**, **Cu-TMPc**, **Zn-TMPc** according to the order. Overall, the nonlinear optical susceptibility [χ^(3)^] has also been determined to be in the order of ∼10^−10^e.s.u. All the detailed estimations of the NLO parameters have been tabulated in Table 2.

**TABLE 2:** Summary of the NLO coefficients of phthalocyanines studied in this work in solution form. Femtosecond MHz pulses at 800 nm were used for these investigations.

Excitation at 800 nm with varied input intensity	β×10^−6^ (cm.W^−1^)	σ_2_ (GM)×10^8^	Im [χ^(3)^]×10^−9^ (e.s.u.)	n_2_×10^−11^ (cm^2^/W)	Re [χ^(3)^]×10^−10^ (e.s.u.)	Total [χ^(3)^]×10^−10^ (e.s.u.)
**TMPc**						
1 mW	3.8	3.14	1.8	3.62	27.9	33.6
2 mW	13	5.4	5.3	−	−	−
3 mW	42	17.3	17.2	−	−	−
**Cu-TMPc**						
1 mW	4	1.6	1.65	3.5	22.7	27.9
2 mW	9	3.7	3.72	−	−	—
3 mW	11	4.2	4.1	−	−	—
**Zn-TMPc**						
1 mW	4.2	1.7	1.9	5.7	36.7	40.6
2 mW	24	10	9.8	−	−	−
3 mW	140	57	57.8	−	−	−
**DCM solvent**	0.008	−	0.003	0.3	2.4	2.42

**Excitation (800 nm) and input powers**	**β×10** ^**−10**^ **(cm.W** ^**-1**^ **)**	**σ**_**2**_ **(GM)×10** ^**3**^	**I**_**s**_ **(W/cm** ^**2**^ **)×10** ^**9**^	**Im [χ** ^**(3)**^ **]×10** ^**−14**^ **(e.s.u.)**	**n**_**2**_ **×10** ^**−16**^ **(cm** ^**2**^ **/W)**	**Re [χ** ^**(3)**^ **]×10** ^**−14**^ **(e.s.u.)**	**Total [χ** ^**(3)**^ **] × 10** ^**−14**^ **(e.s.u.)**
**TMPc**							
0.4 mW	2.3	9.3	6	9.2	−	−	−
0.6 mW	0.8	3.3	15	3.3	4.9	3.2	4.57
0.9 mW	0.5	2	8	2.04	−	−	−
**Cu-TMPc**							
0.4 mW	1.1	4.5	−	4.5	−	−	−
0.6 mW	3.8	15	−	15.5	4.7	4.6	15.9
0.9 mW	5	20.6	−	20.4	−	−	−
**Zn-TMPc**							
0.4 mW	14	57	1	57.3	−	−	−
0.6 mW	5	21	3	20.4	7.1	3.1	20.9
0.9 mW	1.5	6.2	5	6.1	−	−	−
**DCM solvent**	0.008	−	−	0.03	0.11	0.08	0.09

High repetition rate fs, MHz pulses generally have contribution from thermal nonlinearity, leading to higher NLO coefficients of the system extracted from Z-scan data. To access the actual/electronic nonlinearity, we have performed the same measurements with the already mentioned solutions utilizing 50 fs, 1 kHz pulses of 800 nm single beam. For, **TMPc** and **Zn-TMPc**, the study has revealed an exotic “M-shaped” open aperture Z-scan data, displaying combined nature of reverse saturation absorption (RSA) and saturation absorption (SA). For initial intensities, the samples depicted SA and for higher peak intensities it switched to RSA. Occurrence of SA shifting/switching to RSA phenomenon has been noticed in several works recently (Allu et al., 2019). With decreasing intensity, there has been a significant reduction in the nonlinear absorption coefficient, whereas a clear rise in SA nature has been pointed out. We have evaluated the saturation intensity (I_s_) where we observed this “M-shaped” behavior. In the case of **Cu-TMPc**, pure RSA nature has been observed, with decrease in transmission with increasing peak intensities. This kind of behavior was not observed with MHz fs pulses at 800 nm and this could possibly be explained based on the spectral bandwidth of both the pulses used. In the kHz case, the pulses were broad enough with a typical FWHM of >25 nm and an overall spectral width of >50 nm whereas in the case of MHz pulses, they had a FWHM of 8-10 nm with an overall spectral width of 25 nm. These molecules depicted small absorption close to 800 nm and the kHz pulses were able to access this small absorption and this resulted in saturation at initial (low) peak intensities. For higher peak intensities, the SA was overcome by the RSA as we have observed. These promising properties make these compounds potential candidates for optical switching applications. In the case of closed aperture Z-scan data, all three phthalocyanines have illustrated a focusing Kerr lens effect, revealing a downfall in the transmittance followed by a rise in it.

**FIGURE 5:**
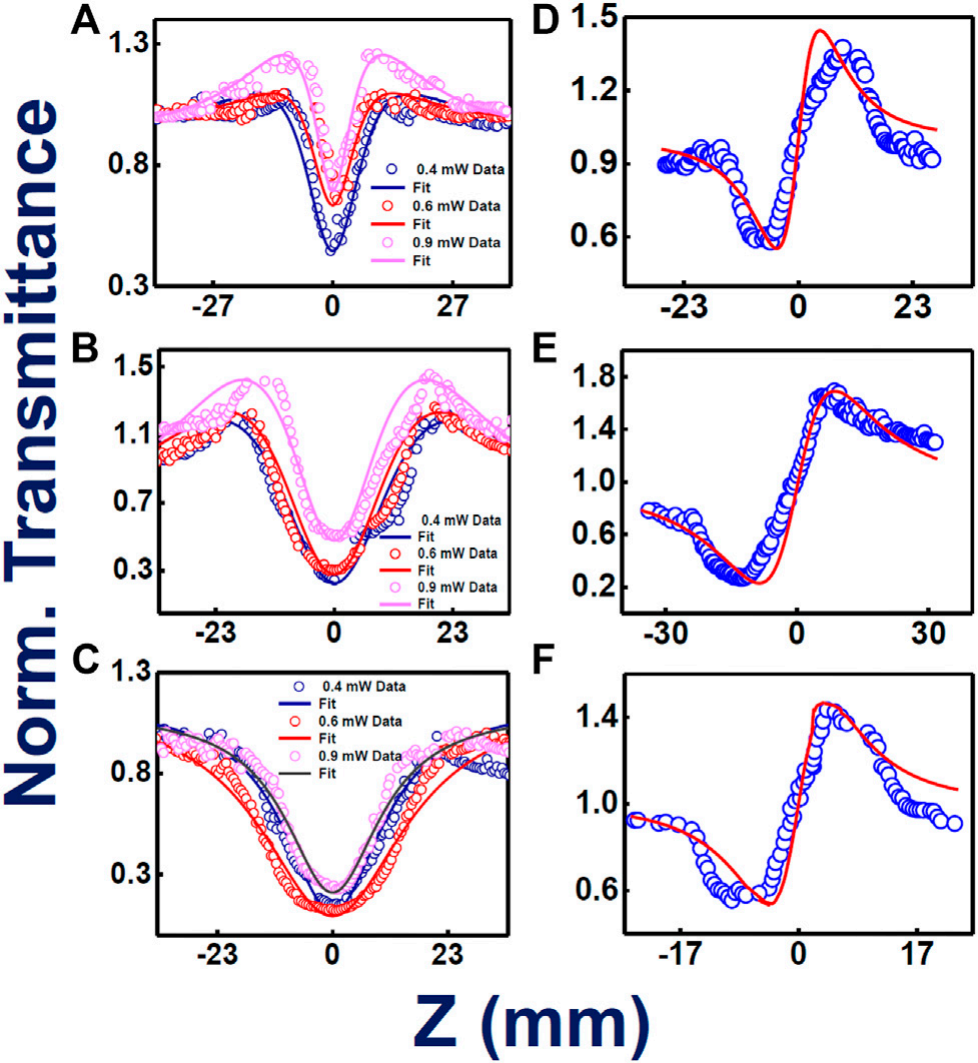
Femtosecond kHz pulses involved experimental and theoretically fitted Z-scan data (intensity dependent) open aperture **(A–C)** and closed aperture configurations **(D–F)** for **TMPc**, **Zn-TMPc, Cu-TMPc**, respectively, in solution form. Open symbols are the experimental data while the solid lines represent theoretical fits.

Figures 5A–C provide the information about the open aperture data of phthalocyanines **TMPc**, **Cu-TMPc**, **Zn-TMPc,** respectively, with a gradual variation in peak power/intensity. The peak intensities where the measurement has been performed were within the range of 400–600 GW/cm^2^. In the case of kHz pulses, peak intensities turned out to be much higher compared to MHz pulses but we were sure of probing only the electronic nonlinearity because of the repetition rate. At the lowest peak intensity, two-photon absorption coefficients (β) value for **TMPc**, **Cu-TMPc**, **Zn-TMPc** revealed themselves to be 2.3 × 10^−10^ cm.W^−1^, 14 × 10^−10^ cm.W^−1^, and 1.1 × 10^−10^ cm.W^−1^ and the corresponding saturation intensity (I_s_) turned out to be 6 × 10^9^ W/cm^2^, 1 × 10^9^ W/cm^2^ for sample without central element (**TMPc**) and “Zn” central element incorporated phthalocyanine, respectively. In the case of pure RSA nature for **Cu-TMPc** molecules, the β values have been found to be in the range of (1-5)×10^−10^ cm.W^−1^. In this study also, **Zn-TMPc** has demonstrated superior (nonlinear) two-photon absorption compared to the other two molecules. Evidently, TmPc and Zn-TmPc data conspicuously unveiled a decreasing nature in their TPA coefficient with increasing input power of the laser excitation, we believe that the reason behind this is the inherent occurrence of stronger bleaching with growing laser excitation intensity, leading to prominence in enhanced transparency, i.e., saturation absorption (SA). Whereas, in the case of Cu-TmPc, the reason behind upsurge in the TPA coefficient with intensity can possibly be attributed to the near resonant excitation circumstances (800 nm laser excitation and two-photon states above 400 nm being available as evident from the absorption spectra). It is pertinent to note that in the case of TmPc and Zn-TMPc, an additional parameter of I_s_ was used in the fittings along with β.

The NLO absorption cross sections (σ_2_) has been extracted and found to be ∼10^3^ GM. Figures 5D–F exhibit the closed aperture Z-scan data, clarifying the n_2_ values to be 4.9 × 10^−16^, 7.1 × 10^−16^, 4.7 × 10^−16^ cm^2^/W for without any component, and Cu, Zn incorporated central cavity phthalocyanines, respectively. The total χ^(3)^ has been calculated of the order of 10^−14^ e.s.u., **Zn-TMPc** having the highest value of 20.9 × 10^−14^e.s.u. Table 2 summarizes all the extracted NLO parameters in detail. The variation in the magnitude of NLO coefficients (Zn-TmPc > TmPc > Cu-TmPc) can possibly be explained in terms of the fine differences in the absorption mechanisms and the excited state lifetimes. Cu-TmPc being non-fluorescent renders the population in the excited state for a shorter time (∼few ps) whereas the other two being fluorescent molecules have lifetimes of the first excited state in the ns regime. Furthermore, TmPc has a slightly stronger absorption in the 800 nm vicinity and saturation effects are expected to be dominant at lower input intensities. In the case of Zn-TmPc, the second excited states are observed at lower energies (below 450 nm) whereas in the Cu-TmPc case they start below 400 nm. The two-photon excitation will be resonant in the case of Zn-TmPc and, therefore, expected to depict a higher NLO coefficient compared to the Cu case. Additional five-level modeling (Rao et al., 1998) along with all the details of excited state dynamics is required to completely decipher the nonlinear absorption mechanisms.

**FIGURE 6:**
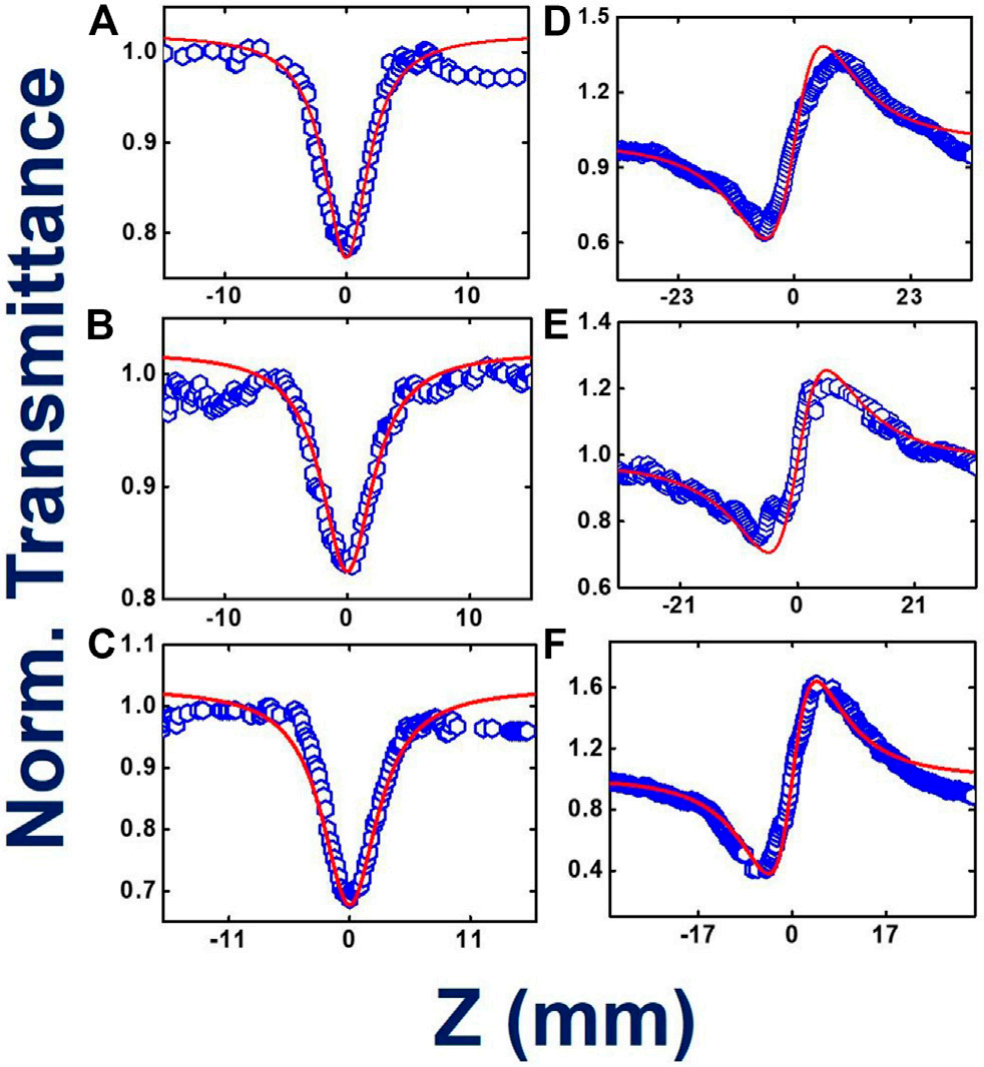
Femtosecond MHz pulses involved experimental and theoretically fitted Z-scan data in open aperture **(A–C)** and closed aperture configurations **(D–F)**, for **TMPc**, **Cu-TMPc**, **Zn-TMPc**, respectively, in thin film form. Open symbols are the experimental data while the solid lines represent theoretical fits.

In Figures 6A–C, we present the fs, MHz open aperture data for thin films with a corresponding peak intensity of ∼15 MW/cm^2^. The β values obtained for the phthalocyanine films **TMPc**, **Cu-TMPc**, and **Zn-TMPc,** respectively, were 2.3 × 10^−7^ cm.W^−1^, 1.7 × 10^−7^ cm.W^−1^, and 3.7 × 10^−7^ cm.W^−1^. Subsequently, their corresponding TPA cross section values (σ_2_) have been determined as 0.9 × 10^7^ GM, 0.7 × 10^7^ GM, and 1.5 × 10^7^ GM. In Figures 6D–F, closed aperture Z-scan data have been illustrated. The extracted magnitudes of n_2_ from the theoretical fits to experimental data turned out to be 1.85 × 10^−11^ cm^2^.W^−1^, 1.1 × 10^−11^ cm^2^.W^−1^, and 2.6 × 10^−11^ cm^2^.W^−1^ according to the same order of molecules. Finally, the order of magnitude of NLO susceptibilities [χ^(3)^] have been found to be ∼10^−10^e.s.u. The nonlinear absorption coefficients in thin films were an order of magnitude lower than in solution, while the n_2_ values were found to be similar in magnitude. This could possibly be attributed to the change in absorption spectra in thin films. Table 3 presents all the details of the measured NLO parameters.

**TABLE 3:** Summary of the NLO coefficients of phthalocyanine thin films studied at 800 nm using 150 fs, 80 MHz pulses.

Excitation at 800 nm with average input power	β×10^−7^ (cm.W^1^)	σ_2_ (GM)×10^7^	Im [χ^(3)^]×10^−10^ (e.s.u.)	n_2_×10^−11^ (cm^2^/W)	Re [χ^(3)^]×10^−10^ (e.s.u.)	Total [χ^(3)^]×10^−10^(e.s.u.)
**TMPc**						
1 mW	2.3	0.9	0.9	1.85	11.8	11.9
**Cu-TMPc**						
1 mW	1.7	0.7	0.74	1.1	7.3	7.3
**Zn-TMPc**						
1 mW	3.7	1.5	1.5	2.6	16.7	16.8
**Glass slide**	0.09	−	0.04	−	−	−

To completely understand and confirm the origin of these fine optical nonlinearities, in each case, the NLO contribution from the corresponding solvents and/or base material (bare glass slide in thin films case) have been verified sincerely by recording their Z-scan data and they have been found to possess negligible magnitude (see Supplementary Figure S20 of the SI file for the data). In both the cases, the contribution of the substrate/solvent was found to be at least 100/10 times smaller than the contribution of the solution/film and, therefore, was ignored. The detailed data have been incorporated in the SI. For better understanding, a clear comparison has been presented in tabular form in Table 4, where we have clearly pointed out that the reported novel phthalocyanine molecules in this recent work have exhibited significantly higher NLO properties, making them superior candidates for various optical applications. Throughout these detailed studies and the results obtained, it is evident that significant NLO behavior has been noticed in these molecules. As mentioned above, the vital change observed in the open aperture response from MHz to kHz studies can be attributed to the decisive differences in their spectral bandwidth. Essentially so, the fundamental difference in the optical response corresponding to nonlinear refractive index of the three compounds can be explained in terms of the Kerr lensing effect. MHz data depicted peak-valley structure or negative n_2_, due to high repetition rate inducing thermal nonlinearities, whereas the kHz studies depicted valley-peak structure or positive n_2_ in all the molecules (e.g., see Christodoulides et al., 2010 for all the mechanisms responsible for n_2_ in organic molecules). The reason behind the ascending order of magnitude of n_2_ for **Cu-TMPc**, **TMPc**, and **Zn-TMPc** could arise from the respective electronic distribution among the excited states or their orientation when the intense light pulse is incident. The absorption cross sections, imaginary as well as the real parts of the NLO susceptibility, are the fundamental parameters for any novel third-order NLO material developed, and these have given a promising clarity to reach out for a decisive conclusion that **Zn-TMPc** has turned out to be a potential compound by exhibiting significantly superior optical nonlinearity in comparison with **Cu-TMPc** and **TMPc**. Another crucial nature has got revealed corresponding to the **Zn-TMPc** and **TMPc** molecule, from their fine exotic “M curve nature”, as it stands for a tunable/shifting NLO absorption behavior depending on variable peak intensities. In regard of an unwavering structural-nonlinear property observation, usually revealed out from the complete study, “Zn” incorporation in the central cavity of tri-methoxy-phenoxy-phthalocyanine, has seemed to open up a possibility of much superior optical nonlinearity.

**TABLE 4:** Summary of various novel phthalocyanines and their derivatives reported recently and their NLO coefficients.

Compound	Laser parameters	β (cm/W)	σ_TPA_ (GM)	n_2_ (cm^2^/W)	χ^(3)^ (e.s.u)	Solvent	Reference
**Pc1, Pc2**	100 fs, 10 Hz	−	−	∼5.7, 8.4 × 10^−15^	−	DMF	Ma et al. (2010)
	800 nm	—	—	—	—	—	—
**CuPc-doped PMMA**	50 fs, 1 kHz	3.22	−	−	−	Chloroform	Li et al. (2008)
	800 nm	×10^−9^	—	—	—	—	—
**Mn(III)-Phthalocyanine chloride**	50 fs, 1 kHz	(9.5–15)	−	−	−	DMSO	Makhal et al. (2017)
	800 nm	×10^−10^	—	—	—	—	—
**Pyrene-conjugated zinc(II) phthalocyanines**	800 nm,1 kHz	−	−	∼(11-13)×10^−16^ cm^2^/W	−	DMF	Husain et al. (2021)
	90 fs	—	—	—	—	—	—
**Alkoxy phthalocyanines**	800 nm, 100 fs	∼10^−12^	-	∼10^−13^e.s.u	—	Chloroform	Venkatram et al. (2008a)
	1 kHz	—	—	—	—	—	—
**Carbazole induced**	600-800 nm, 70 fs	∼ (0.05–7)	∼5-550	∼(0.4–9)	(1.5–79)	DMF	Bhattacharya et al. (2019b)
**Phthalocyanine derivatives**	1 kHz	× 10^−11^		×10^−16^	×10^−15^		
**Alkyl phthalocyanines**	800 nm, 100 fs,	−	−	∼10^−15^	−	Chloroform	Kumar et al. (2007)
	1 kHz	—	—	—	—	—	—
**Triphenyl imidazole induced phthalocyanine derivative**	600–1500 nm	∼ (2-12)	1,500-7,000	∼(0.1–28)	(1-6)	THF	Bhattacharya et al. (2019b)
	1kHz, 70 fs	×10^−11^	—	× 10^−16^	×10^−15^	—	—
***bis*(3,5-Trifluoromethyl)phenyl-zinc phthalocyanine**	690 nm, 70 fs	∼5.9	−	∼6.8 × 10^−17^	−	THF	Krishna et al. (2015)
	1 kHz	×10^−12^	—	—	—	—	—
**Triphenyl imidazole induced phthalocyanine derivative**	700-900 nm	∼ (2-8)	80,000-480000	−	∼10^−10^	DMF	Bhattacharya et al. (2019a)
	150 fs, 88 MHz	× 10^−8^	—	—	—	—	—
**Zinc phthalocyanine**	700-950 nm	∼ (0.2–7)	∼ 10^7^ GM	−	∼10^−9^	DCM	Bharati et al. (2018)
	150 fs, 80 MHz	× 10^−7^	—	—	—	—	—
**Mn(III)-Phthalocyanine chloride**	40 fs, 94 MHz	∼6 × 10^−8^	−	−	−	DMSO	Makhal et al. (2017)
	800 nm	—	—	—	—	—	—
**Pyrene-conjugated azaphthalocyanines**	90 fs, 1 kHz	Three-photon absorption observed	−	13.49 ± 0.18×10^−16^	−	DMF	Sebastian et al. (2020)
	800 nm	—	—	12.85 ± 0.34×10^-16^	—	—	—
**Phthalocyanineporphyrin conjugates metalated with nickel**	190 fs, 515 nm	2.6 × 10^−13^	−	−	−	DCM	Xiao et al. (2016)
	20 Hz	1.7 × 10^−13^	—	—	—	—	—
**3,4,5-Trimethoxyphenyl substituted**	800 nm, 50 fs,	∼14 × 10^−10^ and (4-140) ×10^−6^	(9,300-57,000) and 10^8^	∼7 × 10^−16^ and ∼10^−11^	20 × 10^−14^ and ∼10^−10^	DCM	This Work
	1 kHz&						
	800 nm, 150 fs						
	80 MHz						

To put the NLO performance in perspective of the studied molecules, we compared the NLO coefficients of title compounds with some of the newly reported phthalocyanine molecules under similar experimental conditions. A similar comparison exercise has been reported by us in our recent work on porphyrins (Kumar et al., 2021b) and phthalocyanines (Bhattacharya et al., 2021). There have been several NLO reports on novel phthalocyanines but with nanosecond pulses (see, e.g., Schmidt and Calvete, 2021), and we have not considered those in this comparison study. Recently a dimeric phthalocyanine zinc complex (bis-[2-hydroxy-9(10), 16(17), 23(24)-tri-*tert*-butylphthalocyanine]zinc–J-[^OH^Pc^t^Zn]_2_) was prepared and the NLO properties were investigated using ∼280 fs pulses (Kazak et al., 2021). They observed two-photon and three-photon absorption and reported coefficients of β_eff_ = 312 cmGW^−1^ and a γ_eff_ = 8.8 cm^3^GW^−2^. Phthalocyanine nanoprobes were reported recently, and they have been demonstrated as highly desirable for photoacoustic molecular imaging (Li et al., 2021). Two-photon absorption coefficients of ∼10^−13^ cm/W were reported for phthalocyanine porphyrin conjugates metalated with nickel by Xiao et al., 2016. Interestingly, n_2_ values with magnitudes of 10^−16^ cm^2^/W were reported recently for pyrene-conjugated azaphthalocyanines (Sebastian et al., 2020) and pyrene-conjugated Zinc(II)phthalocyanines (Husain et al., 2021), respectively. However, they observed strong three-photon absorption in the open aperture data.

Our group has recently reported the NLO properties of two unsymmetrically substituted novel porphycenes, i.e., 2,7,12,13-tetraethyl-9,10,19,20-tetramethyl(OAPo-T) and 2,3,16,17-tetraethyl-9,10,19,20-tetramethyl (OAPo-C) porphycenes with TPA cross-sections of 159 and 145 GM, respectively, obtained with exact experimental conditions (kHz, fs pulses at 800 nm) used in the present study. Similarly, one of our earlier works (Rathi et al., 2020) reported fs, MHz NLO data of novel porphyrins [H_2_TPP(TPA)_2_NO_2_, H_2_TPP(TPA)_2_CHO, ZnTPP(TPA)_2_NO_2_, and ZnTPP(TPA)_2_CHO]. The obtained β values were ∼10^−8^ cm/W, σ_TPA_ values of ∼10^5^ GM, n_2_ values of ∼10^−13^ cm^2^/W, and χ^(3)^ values of ∼10^−11^e.s.u. for those molecules. The coefficients retrieved in our present case (see Table 2) are at least one order higher compared to these and this could possibly be attributed to the bulky substituents at the periphery and/or large π electrons. Our group has again reported fs, kHz NLO data (Bhattacharya et al., 2021) of ImCuPc and ImZnPc (similar central metal ions in the present case) wherein we obtained β values of ∼10^−12^ cm/W, n_2_ values of ∼10^−15^ cm^2^/W (in the present case they were ∼10^−10^ cm/W and ∼10^−16^ cm^2^/W). Another work reported (Kumar et al., 2021a) the fs, kHz NLO data with β values of series of “push–pull” meso-substituted trans-A2BC porphyrins, where A = mesityl, B = phenothiazine(push) and C = o/p-nitrophenyl moiety (pull) and M = 2H, Ni(II), Cu(II), and Zn(II) with coefficients of β∼10^−11^ cm/W, n_2_ ∼10^−15^ cm^2^/W. Katturi et al. (2020b) reported fs, kHz NLO studies of D-π-D porphyrins with coefficients of β ∼10^−11^ cm/W, n_2_ ∼10^−15^ cm^2^/W. The same group (Katturi et al., 2020a) again reported the fs, kHz NLO coefficients of β ∼10^−11^ cm/W, n_2_ ∼10^−15^ cm^2^/W newly synthesized donor-acceptor-based free-base [(C_6_F_5_)_3_] and phosphorus [P-(OH)_2_(C_6_F_5_)_3_] corroles. These data clearly indicate that the present molecules have strong NLO coefficients and potential for photonic applications (e.g., in optical imaging, optical limiting, and optical switching).

## Conclusion

As concluding remarks, we state that we have synthesized a new series of non-aqueous phthalocyanines possessing the 3,4,5-trimethoxyphenyl group at peripheral positions. A thorough NLO investigation with femtosecond MHz and kHz pulses has been carried out, successfully probing into the molecules’ NLO coefficients to figure out their ultrafast interaction response nature. Significant achievements have been obtained in the NLO domain with both MHz and kHz pulse excitations. Some of the highlights are as follows:

• Strong TPA coefficients and cross-sections (kHz NLO coefficients were found to be smaller in magnitude than the MHz coefficients and this is as expected). These coefficients were found to be on par or superior to some of the recently reported other phthalocyanine moieties.
• A complicated nonlinear absorption behavior observed with kHz pulse excitation. This has been explained using the absorption profile and spectral bandwidths of the pulses used.
• Thin film NLO coefficients were also evaluated because for practical applications, these are imperative.

After extensive analysis, promising revelations have been clarified about optical nonlinearity being in deterministic relation molecular structural as well as central cavity components of the corresponding phthalocyanine molecules. This may definitely be an advantage in synthesizing novel phthalocyanine molecules according to desirable and tunable optical nonlinearity.

## Data Availability

The original contributions presented in the study are included in the article/Supplementary Material, and further inquiries can be directed to the corresponding author.
