# Supplementary material for: Optical, Electrochemical, Third-Order Nonlinear Optical Investigations of 3,4,5-Trimethoxy Phenyl Substituted Non-Aqueous Phthalocyanines
Source: Front Chem. 2021 Sep 9;9:713939. doi: 10.3389/fchem.2021.713939 (PMC8458761; doi:10.3389/fchem.2021.713939)
Supplement: Supplementary file 1 [file DataSheet1.docx]

**Supporting Information for**

**Optical, Electrochemical, Third-order Nonlinear Optical Investigations of 2,3,4-trimethoxy Phenyl Substituted Non-aqueous Phthalocyanines**

**K. S. Srivishnu,^a,b^ Dipanjan Banerjee,^c^ Ramya Athira Ramnagar,^a^ Jagannath Rathod,^c^ Lingamallu Giribabu,^a,b,#^ Venugopal Rao Soma^c,*^**

^a^Polymers & Functional Materials Division, CSIR-Indian Institute of Chemical Technology, Tarnaka, Hyderabad 500007, TG, India. # E-mail: [**giribabu@iict.res.in**](mailto:giribabu@iict.res.in)

^b^Academy of Scientific and Innovative Research (AcSIR), Ghaziabad 201002, India

^c^Advanced Centre of Research in High Energy Materials (ACRHEM), University of Hyderabad, Prof. C.R. Rao Road, Hyderabad 500046, Andhra Pradesh, India.

Corresponding Author E-mail: [**svrsp@uohyd.ernet.in**](mailto:svrsp@uohyd.ernet.in) **OR** [**soma_venu@uohyd.ac.in**](mailto:soma_venu@uohyd.ac.in)

| **Table of Contents** | | **Page No.** |
| --- | --- | --- |
| **Figure S1** | ^1^H NMR spectrum of phthalonitrile (1) in CDCl_3_. | S3 |
| **Figure S2** | ^1^H NMR spectrum of phthalonitrile (1) in CDCl_3_. | S3 |
| **Figure S3** | FT-IR spectrum of **TmPc**. | S4 |
| **Figure S4** | MALDI-MS spectrum of **TmPc**. | S4 |
| **Figure S5** | FT-IR spectrum of **Zn-TmPc**. | S5 |
| **Figure S6** | MALDI-MS spectrum of **Zn-TmPc.** | S5 |
| **Figure S7** | FT-IR spectrum of **Cu-TmPc**. | S6 |
| **Figure S8** | MALDI-MS spectrum of **Cu-TmPc** | S6 |
| **Figure S9** | Absorption spectra of **TmPc** in different solvents. | S7 |
| **Figure S10** | Absorption spectra of **Cu-TmPc** in different solvents. | S7 |
| **Figure S11** | Absorption spectra of **Zn-TmPc** in different solvents. | S8 |
| **Figure S12** | Emission spectra in different solvents | S8 |
| **Figure S13** | Cyclic voltammogrammes of phthalocyanines in DCM solvent using 0.1 M TBAP. | S9 |
| **Figure S14** | Optimized structures of phthalocyanines. | S9 |
| **Figure S15** | Isodensity plots of FMOs and the energy values in eV by using the B3LYP method 6-31G (d,p) for **TmPc**. | S10 |
| **Figure S16** | Isodensity plots of FMOs and the energy values in eV by using the B3LYP method 6-31G (d,p) for **CuTmPc**. | S11 |
| **Figure S17** | Isodensity plots of FMOs and the energy values in eV by using the B3LYP method 6-31G (d,p) for **ZnTmPc**. | S12 |
| **Figure S18** | Surface coating thickness data of the Phthalocyanine thin films (a), (b), (c) for TMPC, Cu-TMPC, Zn-TMPC, respectively. | S13 |
| **Figure S19** | Absorption spectra of the thin films of phthalocyanines studied in this work. | S14 |
| **Figure S20** | Z-scan data of open aperture (a, b) and closed aperture (d, e) for pure Dichloromethane (DCM) solvent involving fs MHz, kHz pulses respectively. Whereas, Figure (c, f) represents open and closed aperture data for a clean glass slide in interaction with MHz fs pulses. | S15 |
| **Table S1** | Singlet excited state properties of dyes by B3LYP method and M06-2X function in tetrahydrofuran solvent in PCM model. | S16 |


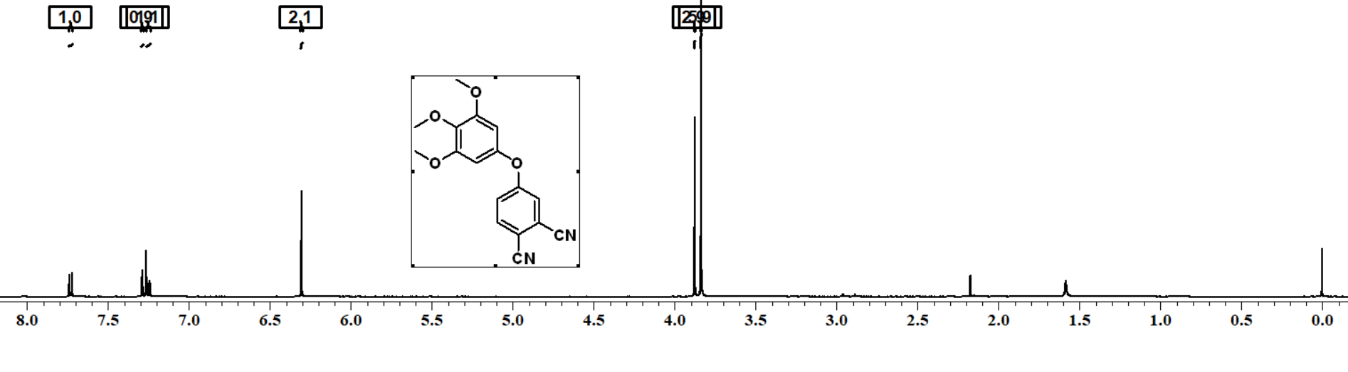


**Figure S1.**^1^H NMR spectrum of phthalonitrile (1) in CDCl_3_.


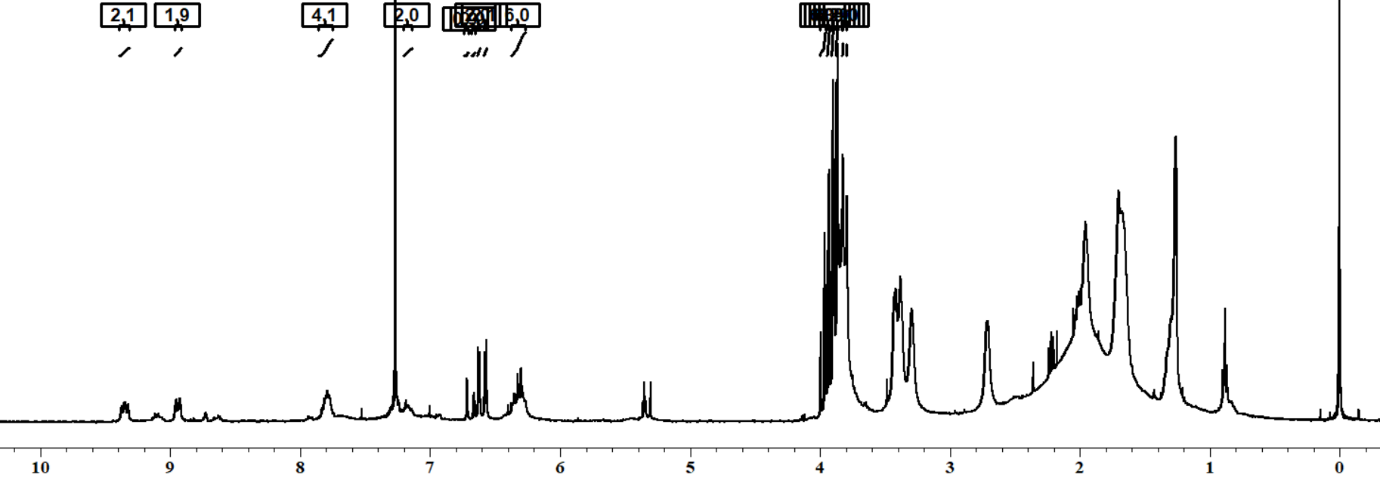


**Figure S2.**^1^H NMR spectrum of phthalonitrile (1) in CDCl_3_.


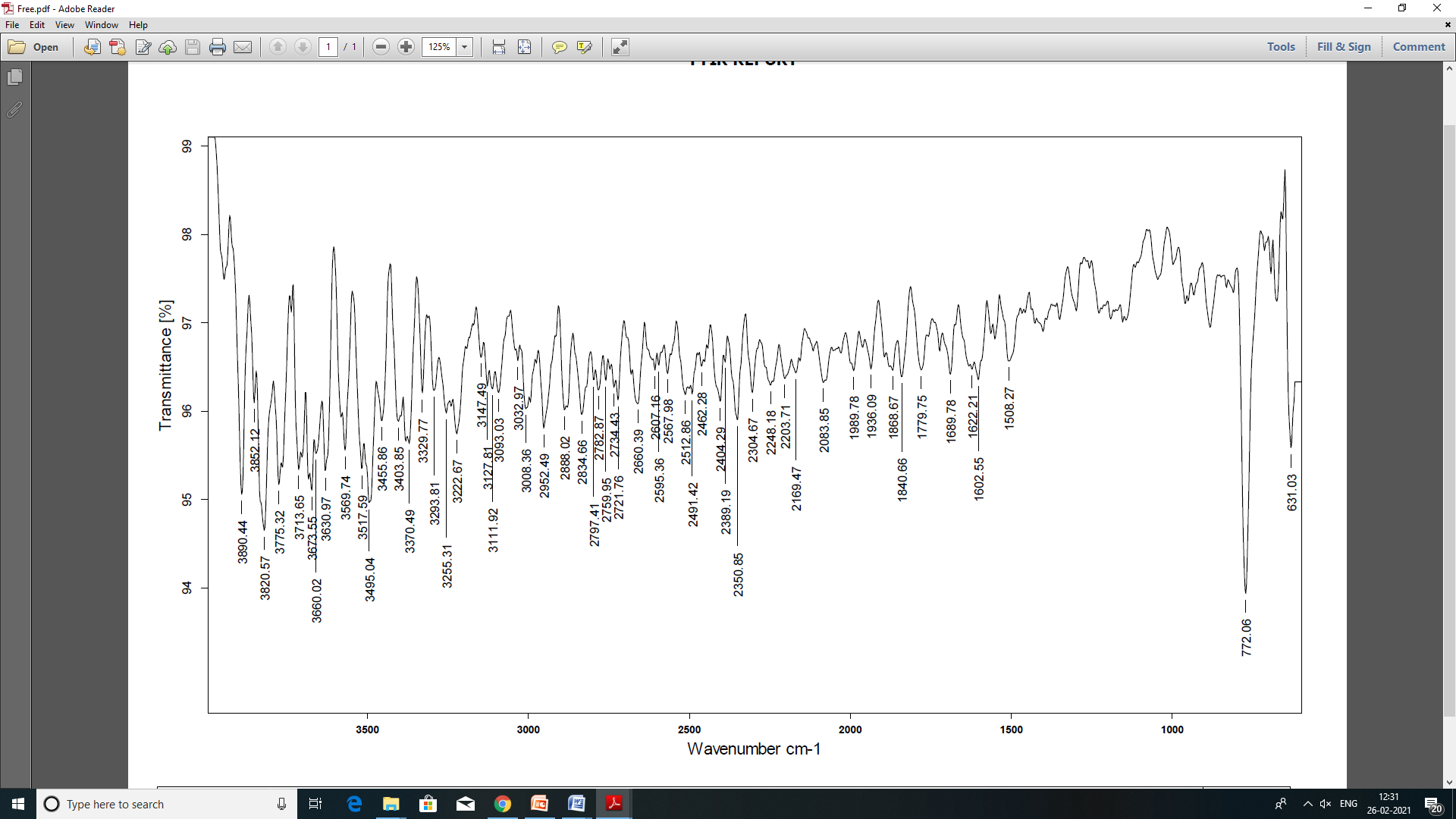


**Figure S3.** FT-IR spectrum of TmPc.


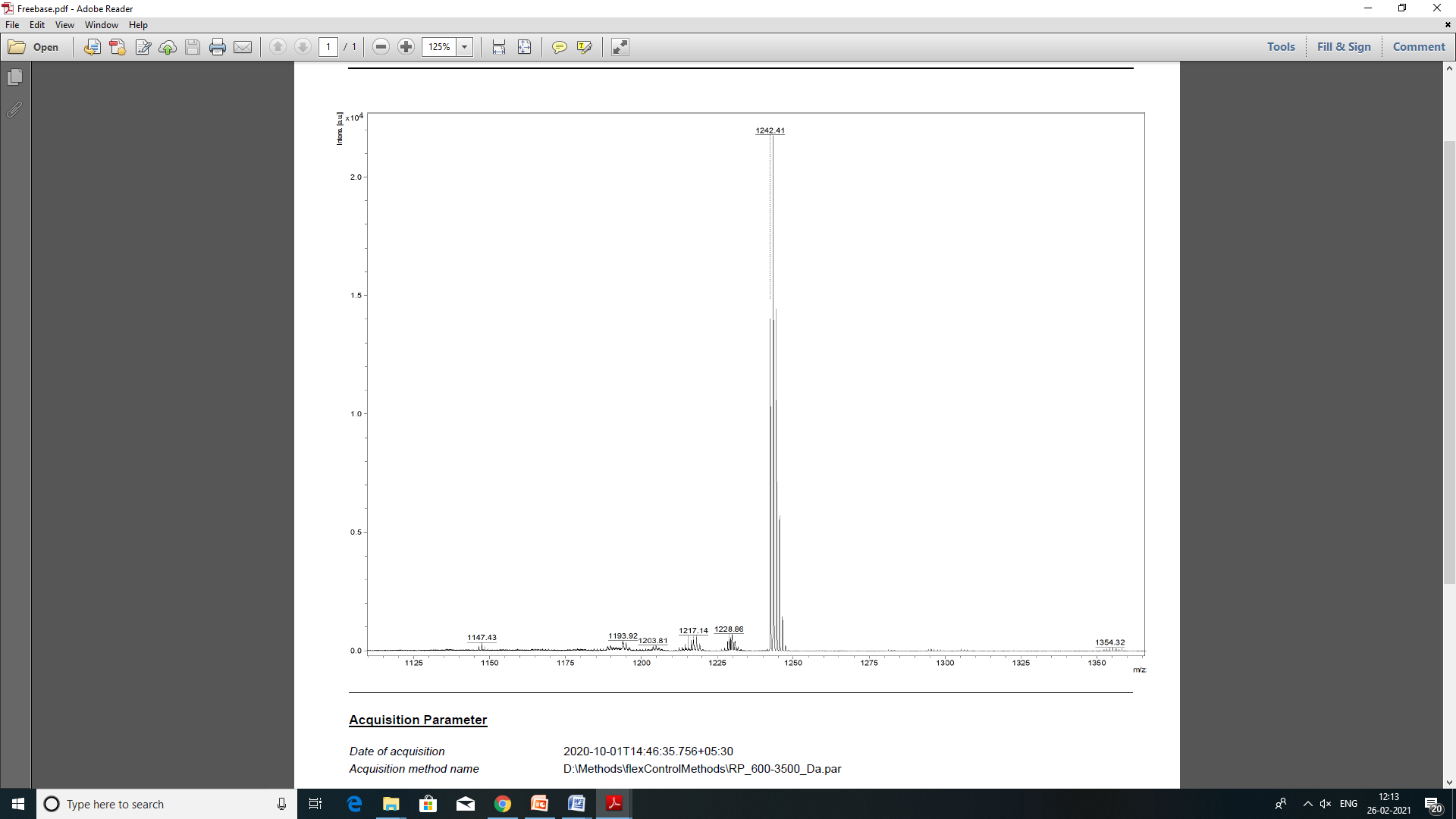


**Figure S4.** MALDI-MS spectrum of TmPc.


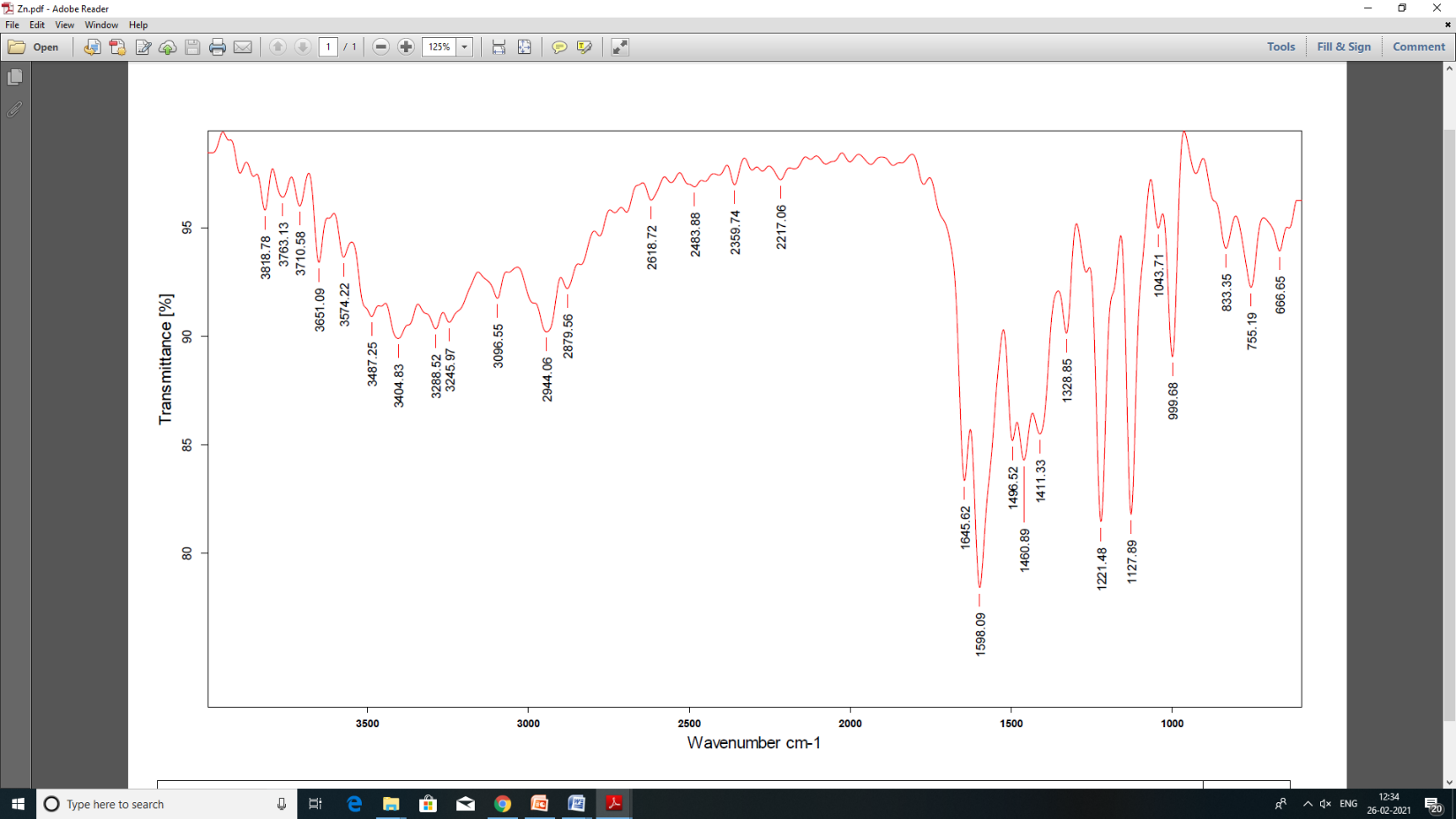


**Figure S5.** FT-IR spectrum of Zn-TmPc.


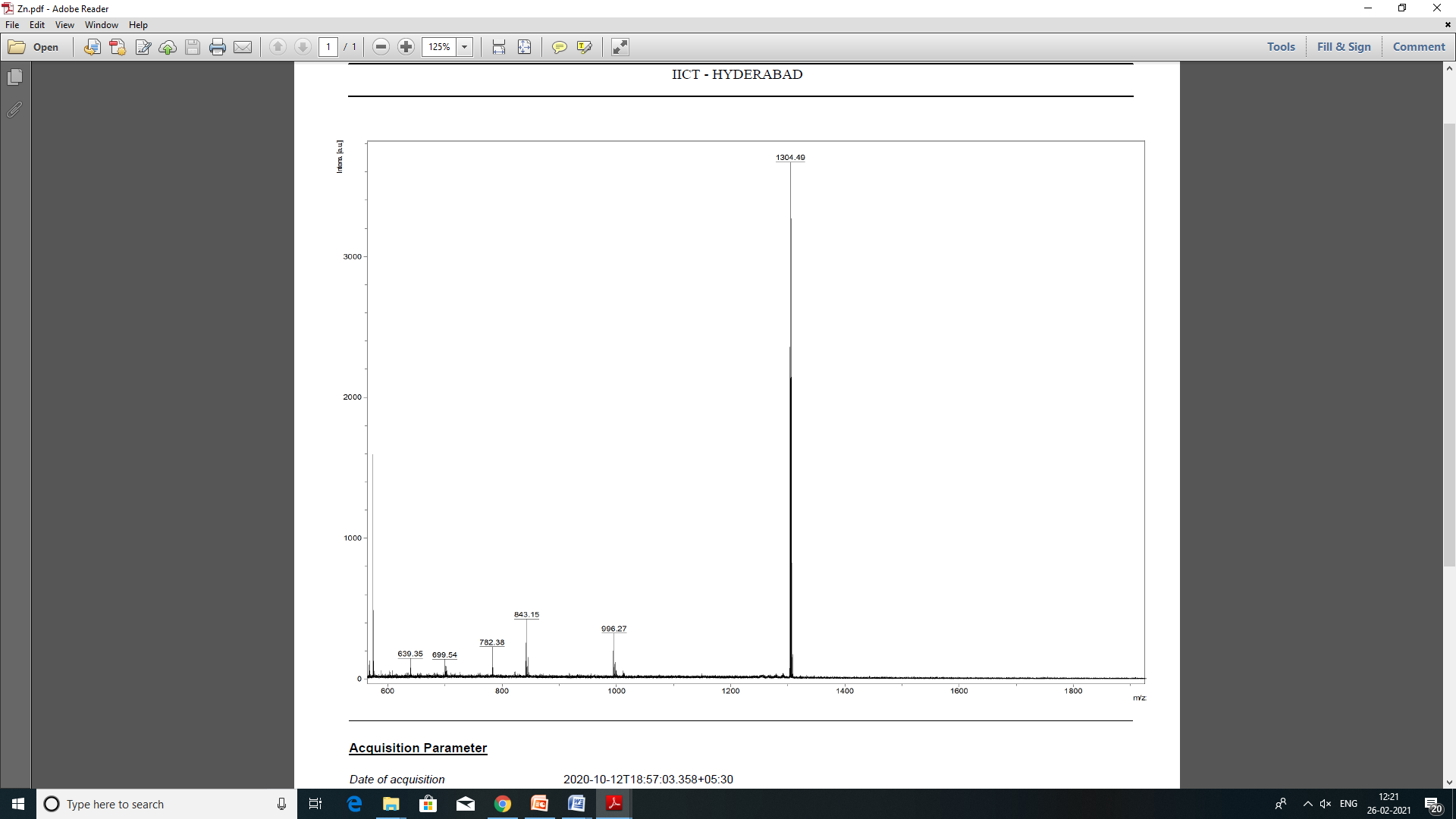


**Figure S6.** MALDI-MS spectrum of Zn-TmPc.


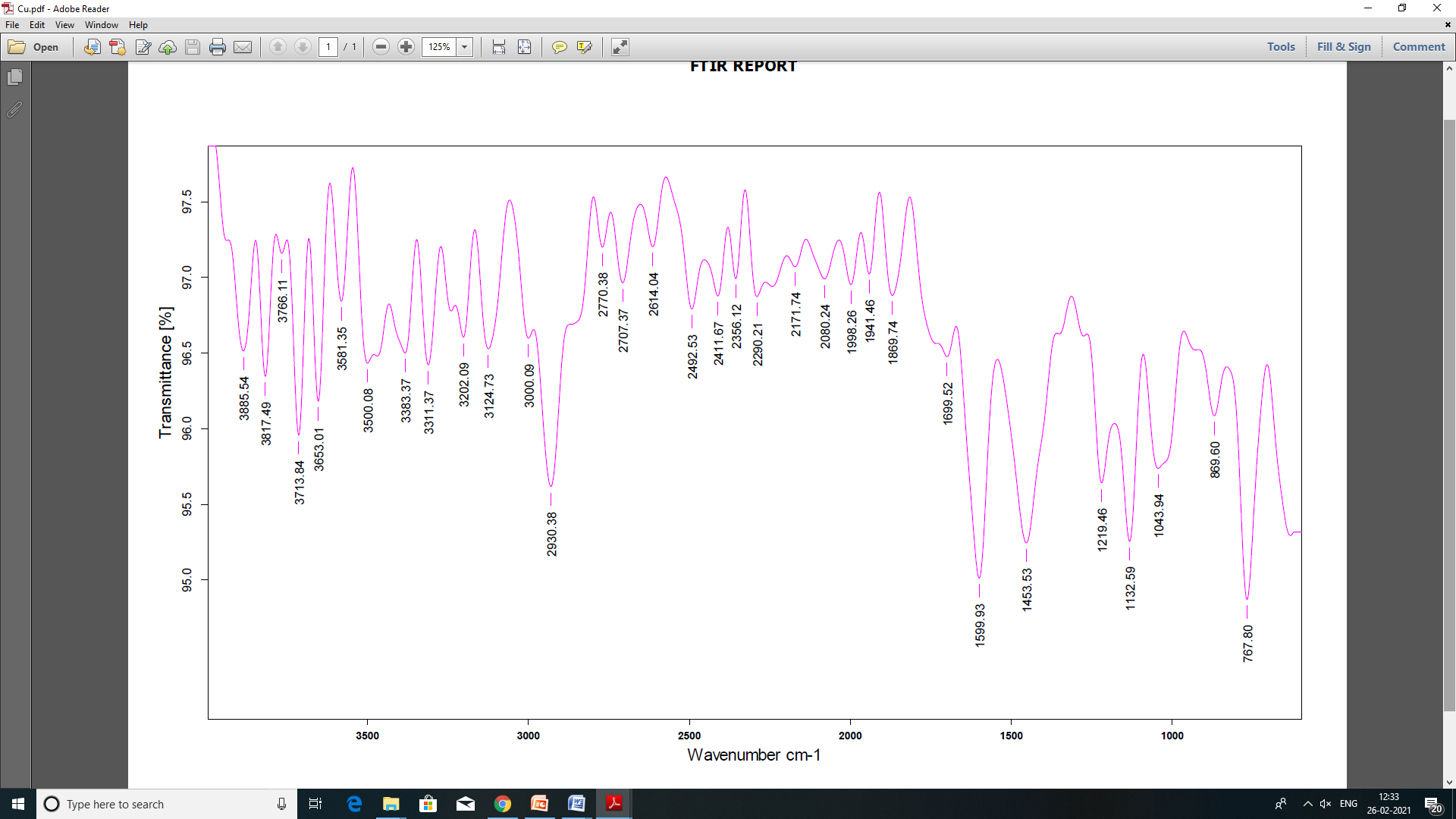


**Figure S7.** FT-IR spectrum of Cu-TmPc.


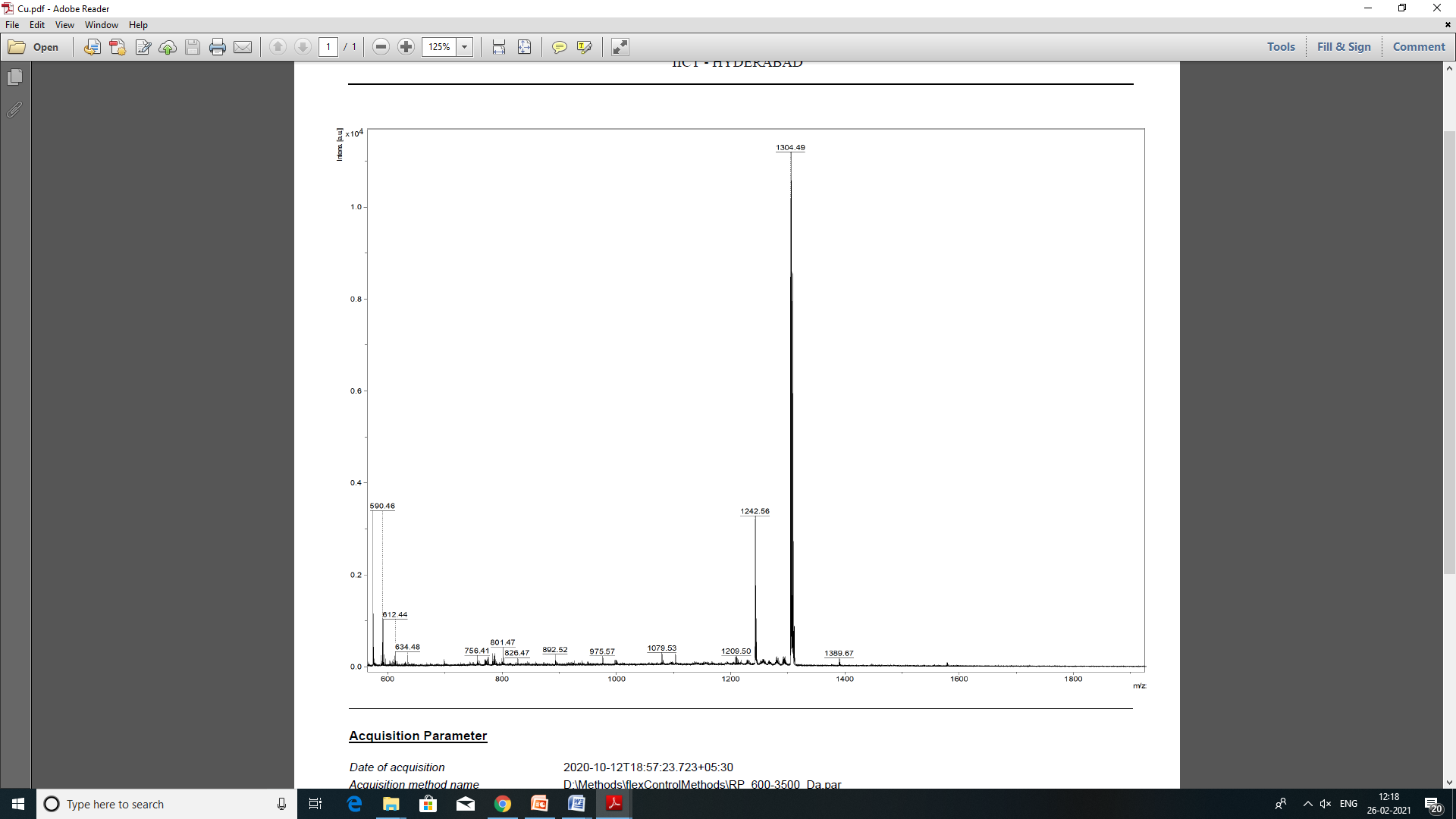


**Figure S8.** MALDI-MS spectrum of Cu-TmPc.

**Figure S9:** Absorption spectra of **TmPc** in different solvents.

**Figure S10**: Absorption spectra of **Cu-TmPc** in different solvents.

**Figure S11**: Absorption spectra of **Zn-TmPc** in different solvents.

**Figure S12.** Emission spectra in different solvents.

^
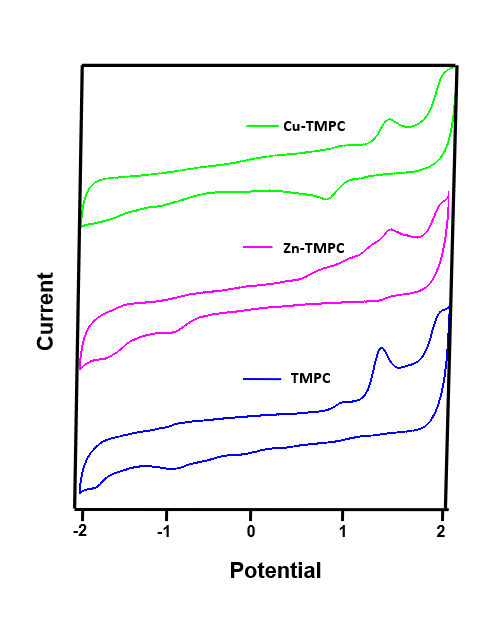
^

**Figure S13**. Cyclic voltammograms of phthalocyanines in DCM solvent using 0.1 M TBAP.


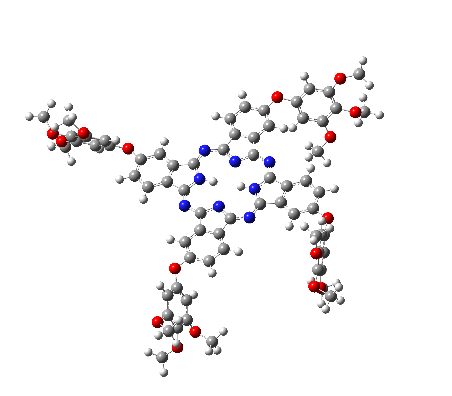

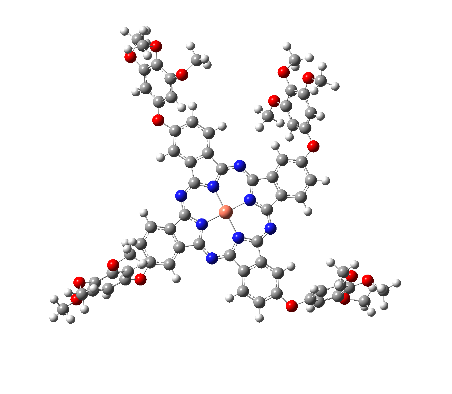

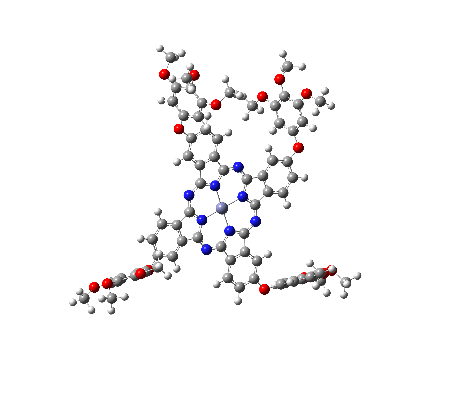


**TmPc CuTmPc ZnTmPc**

**Figure S14.** Optimized structures of phthalocyanines.


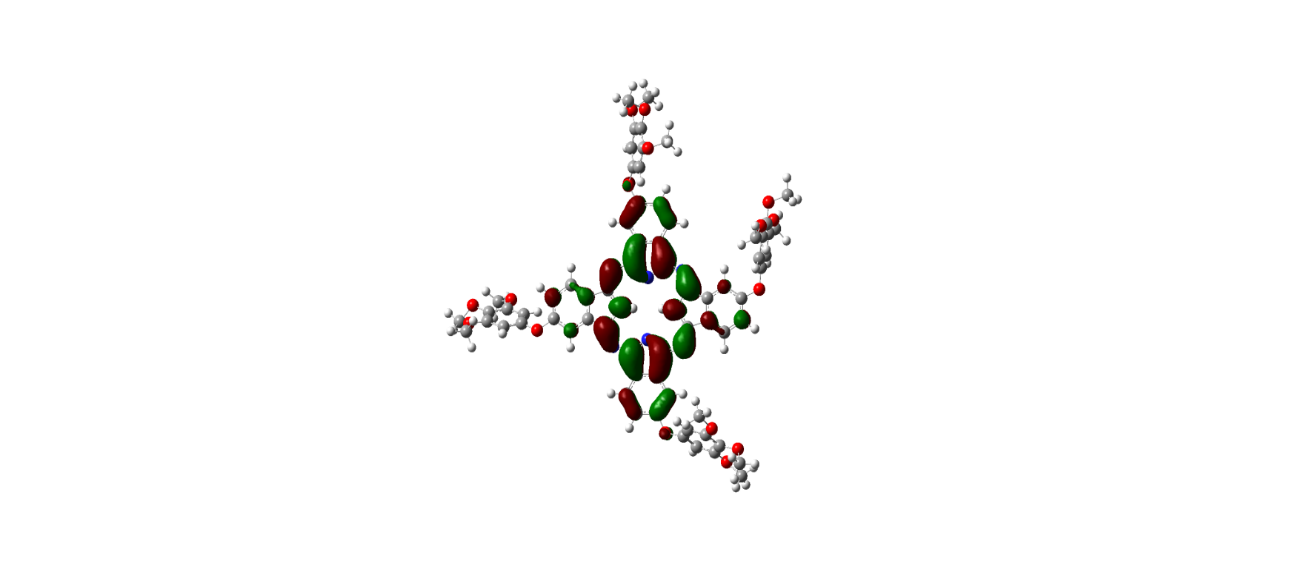

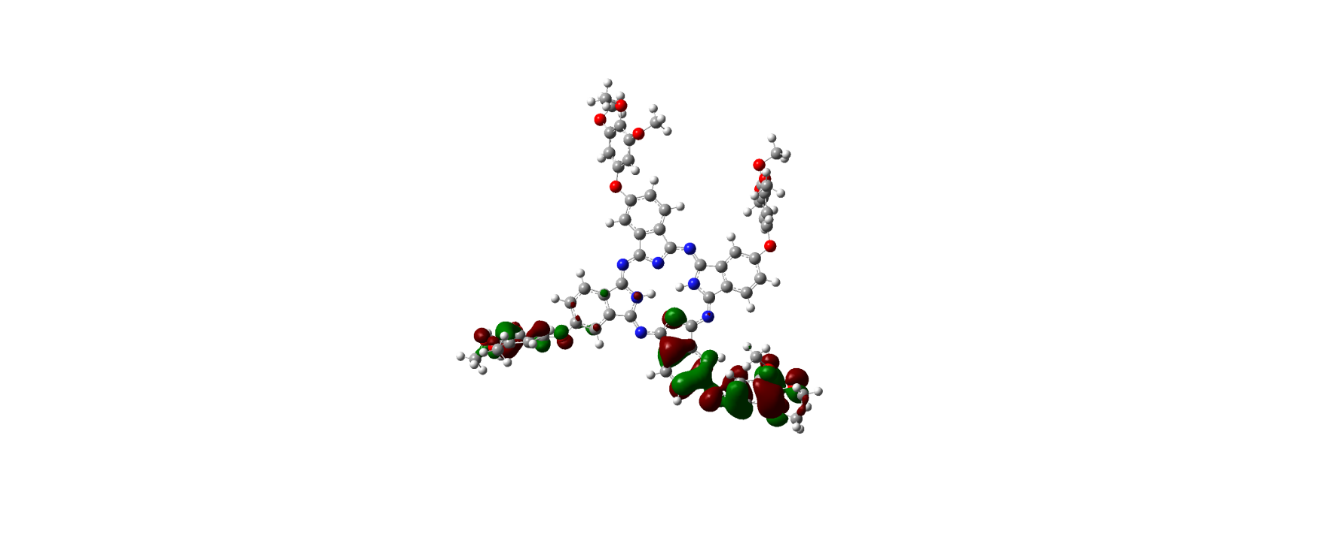

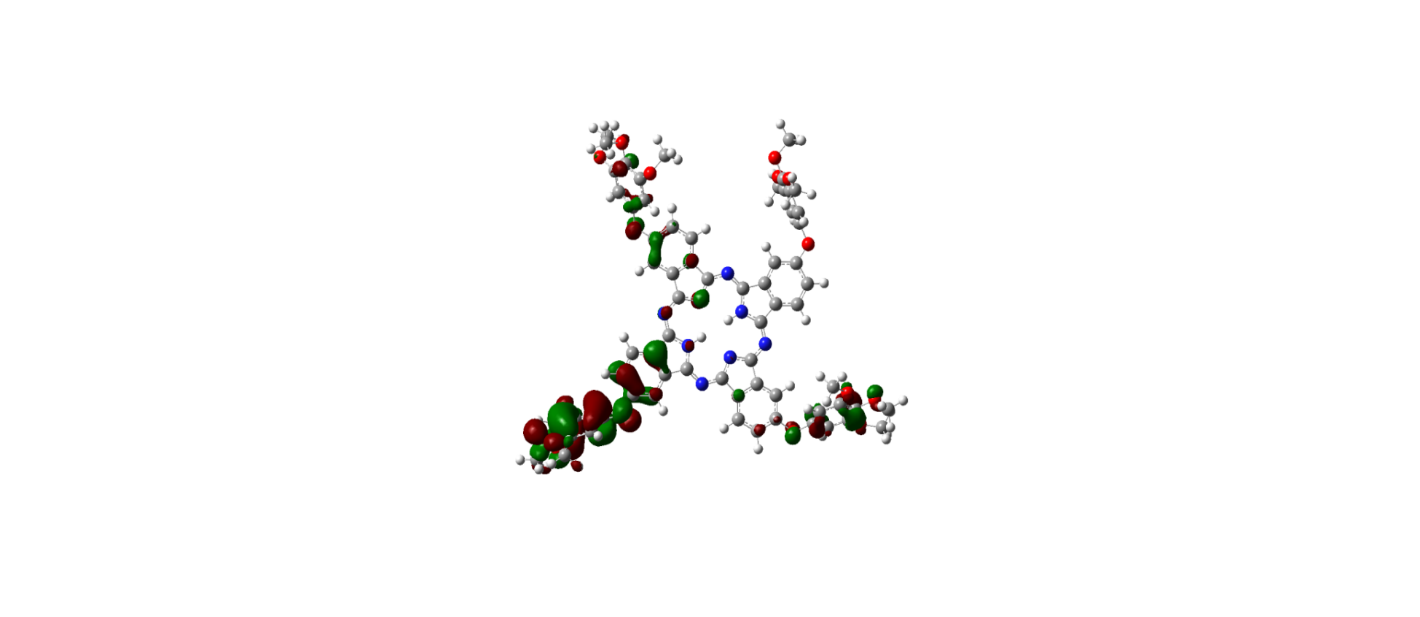


LUMO = -2.66 eV LUMO+1 = -2.60 eV LUMO+2 = 0.95 eV


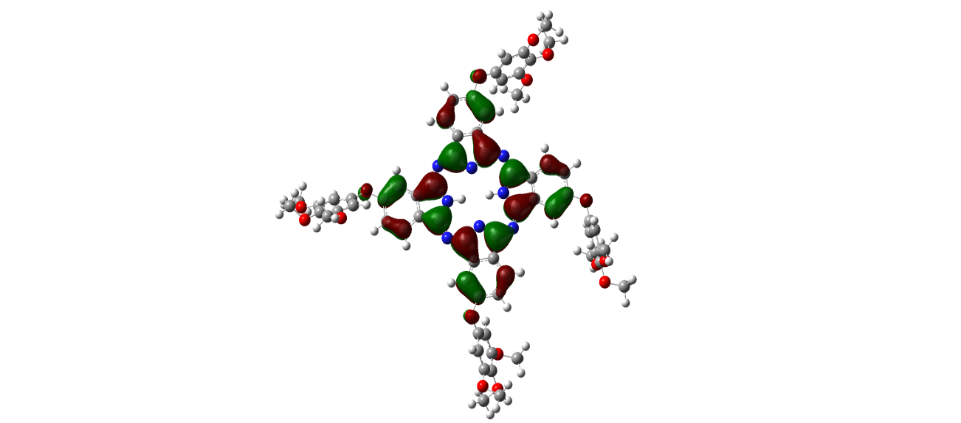

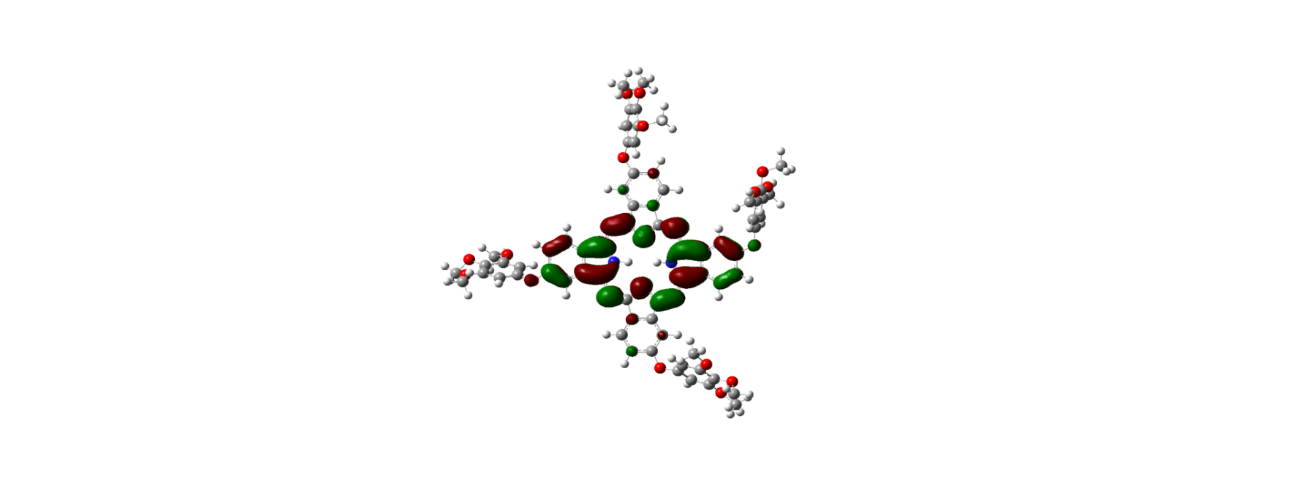

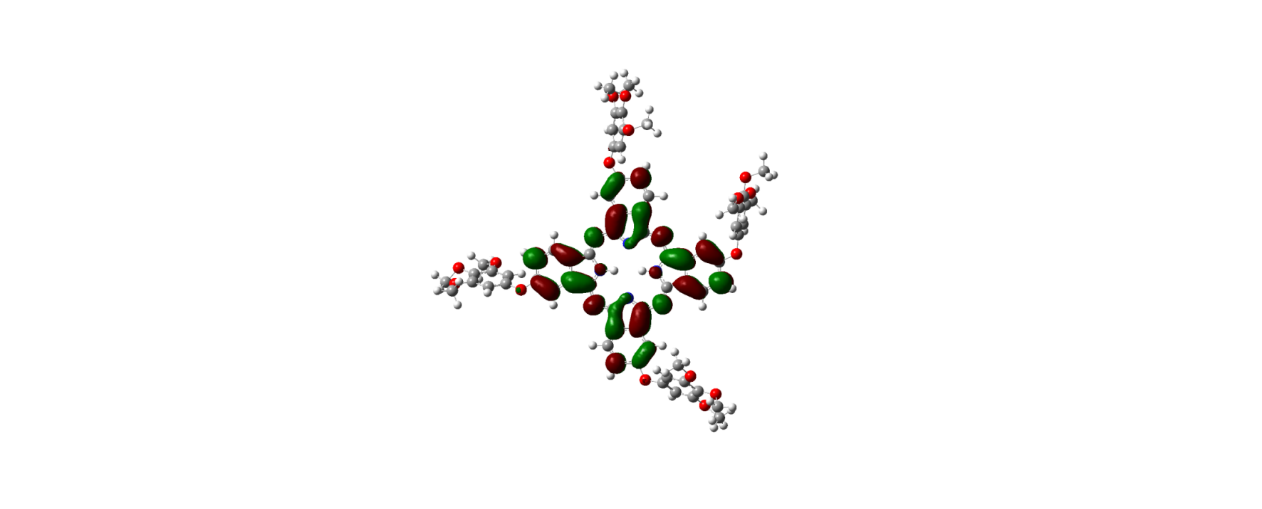


HOMO = -4.76 eV HOMO-1 = -5.60 eV HOMO-2 = -5.62 eV

**Figure S15.**Isodensity plots of FMOs and the energy values in eV by using the B3LYP method 6-31G (d,p) for **TmPc**.


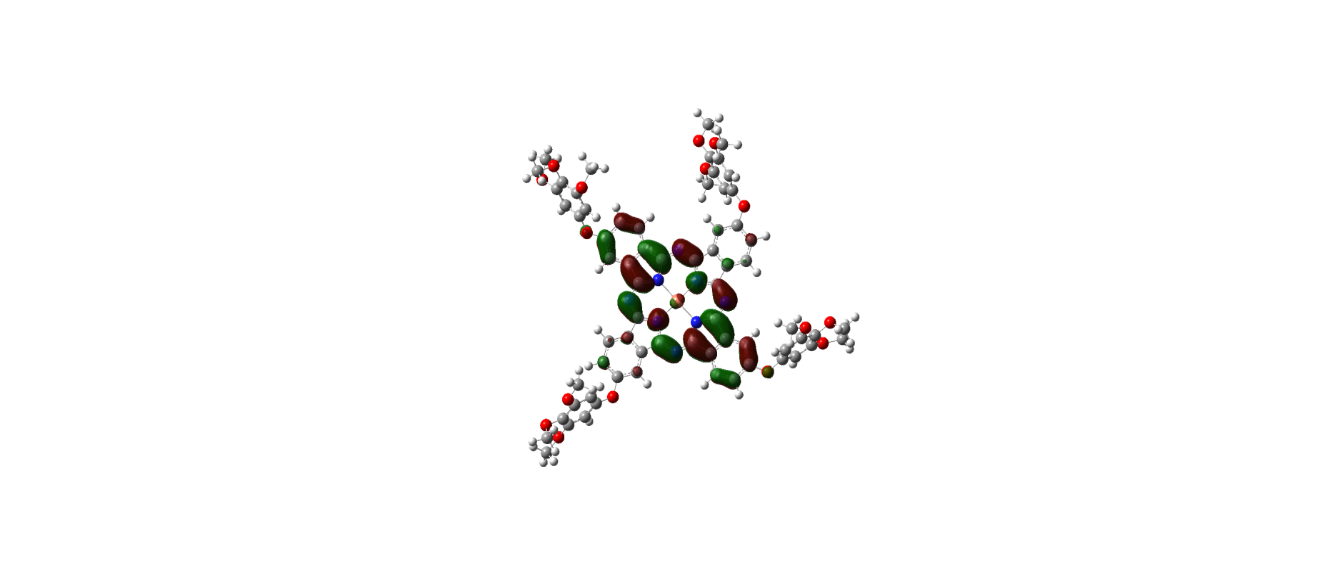

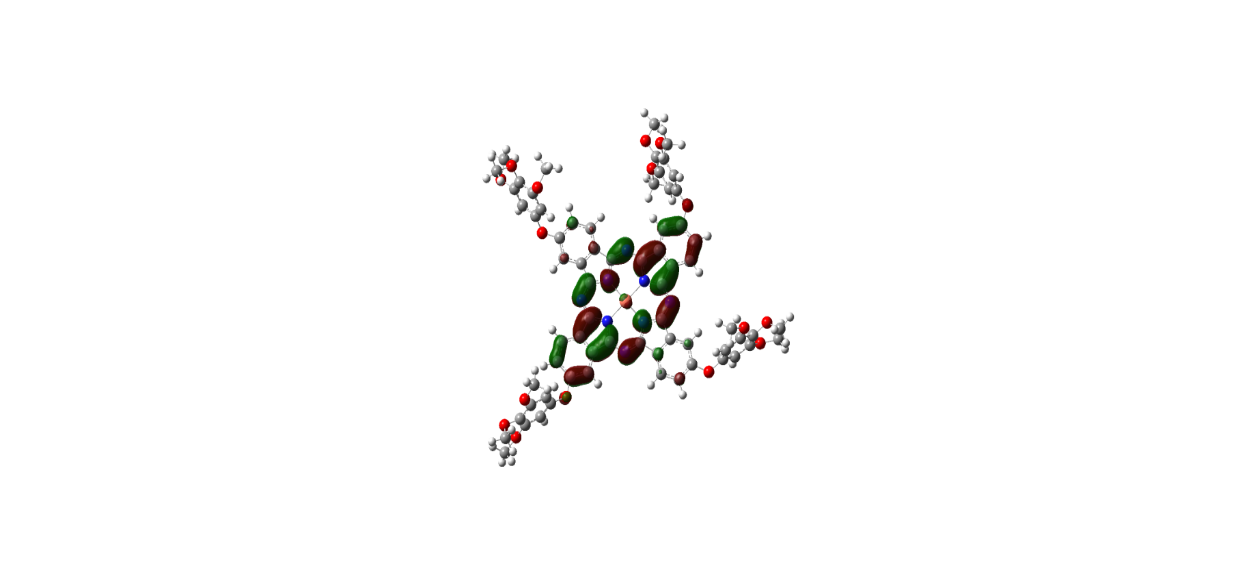

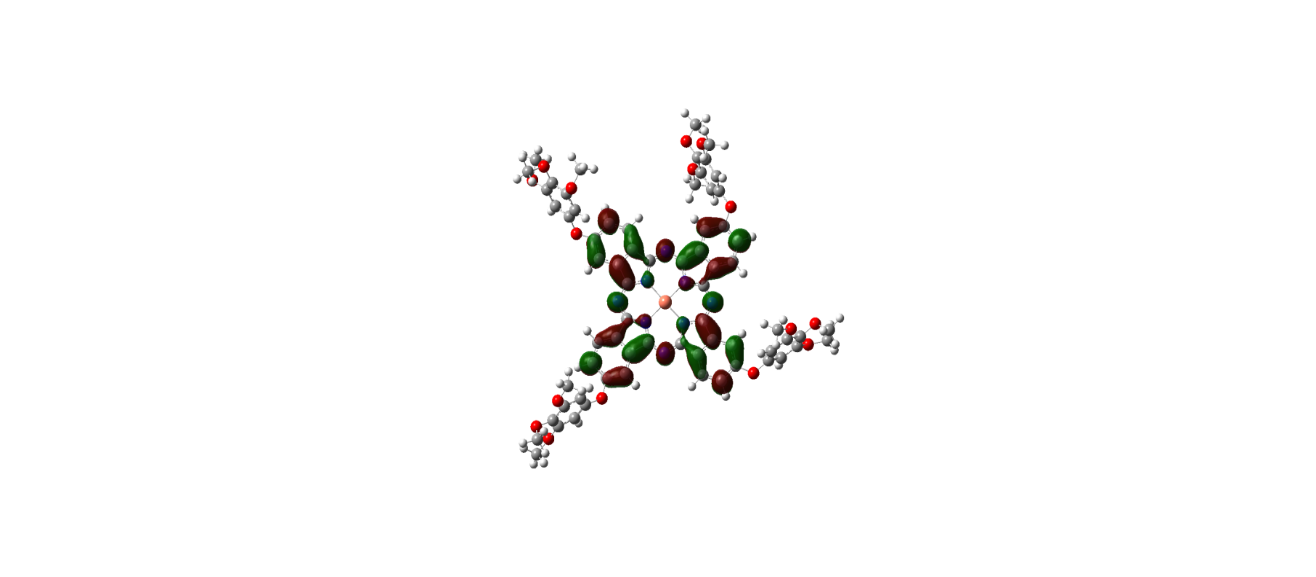


LUMO = -2.59 eV LUMO+1 = -2.58 eV LUMO+2 = 0.93 eV


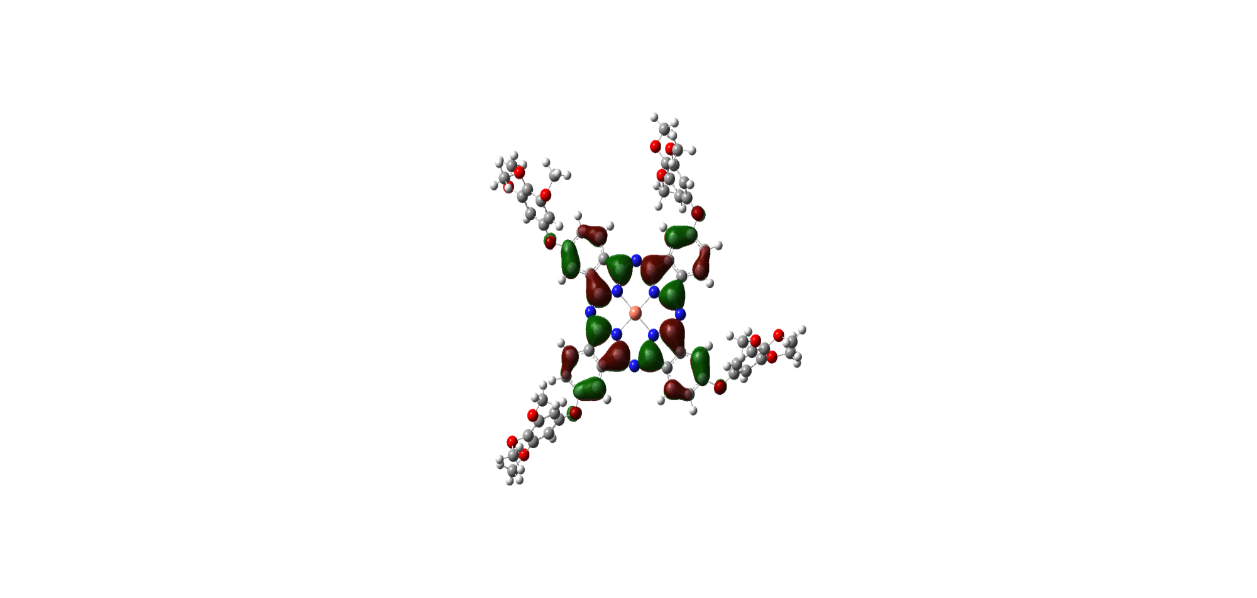

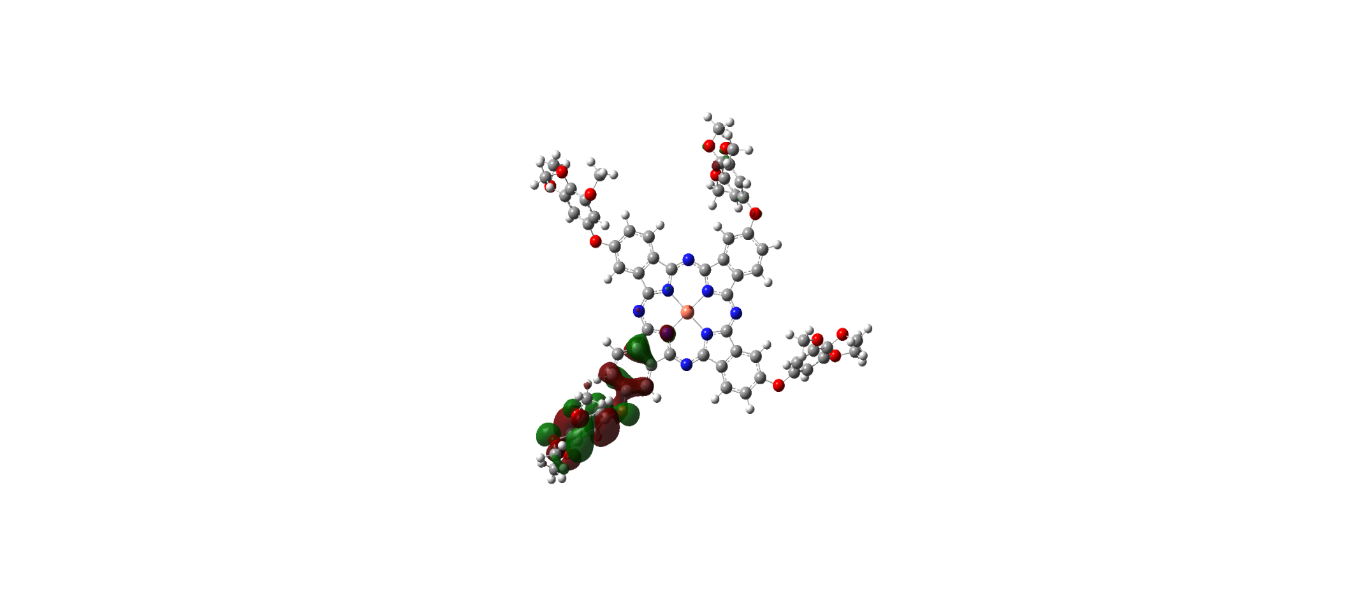

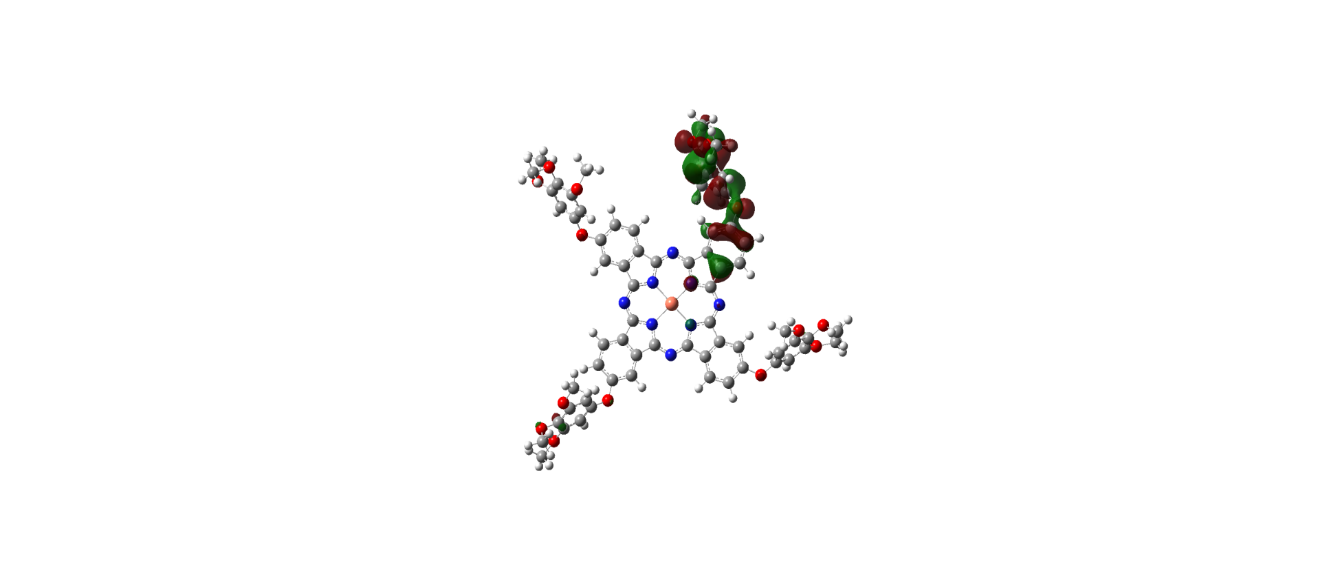


HOMO = -4.75 eV HOMO-1 = -5.63 eV HOMO-2 = -5.70 eV

**Figure S16.**Isodensity plots of FMOs and the energy values in eV by using the B3LYP method 6-31G (d,p) for **CuTmPc**.


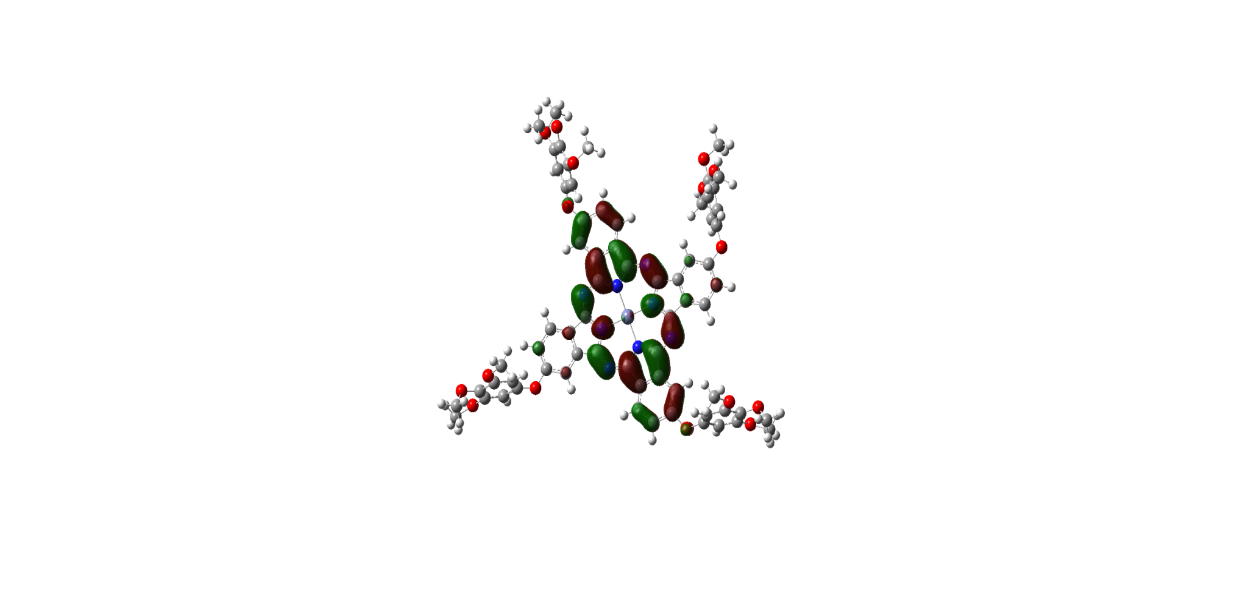

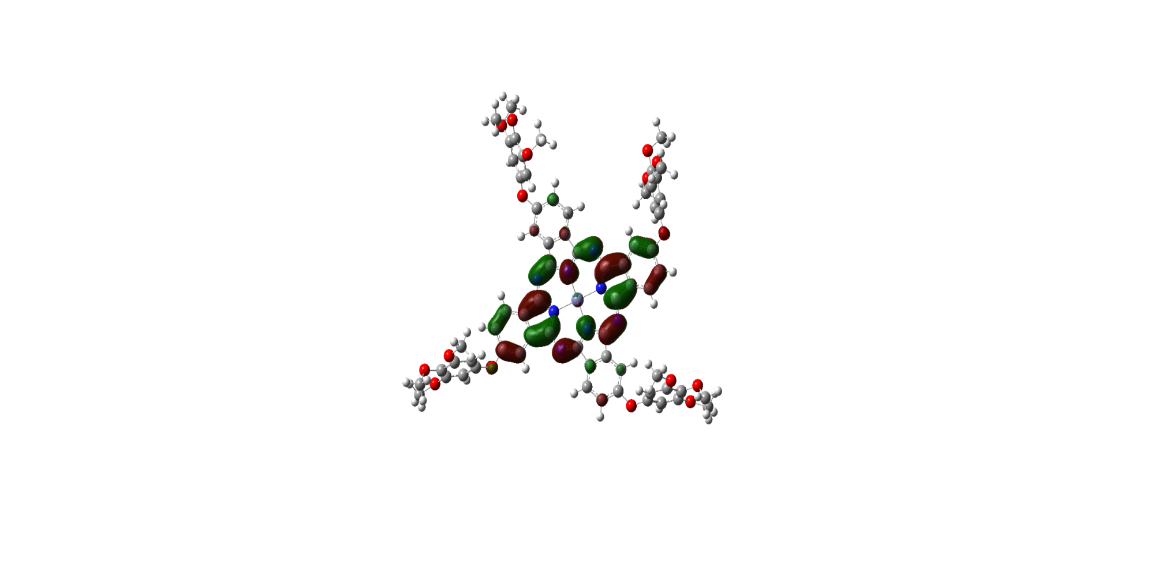

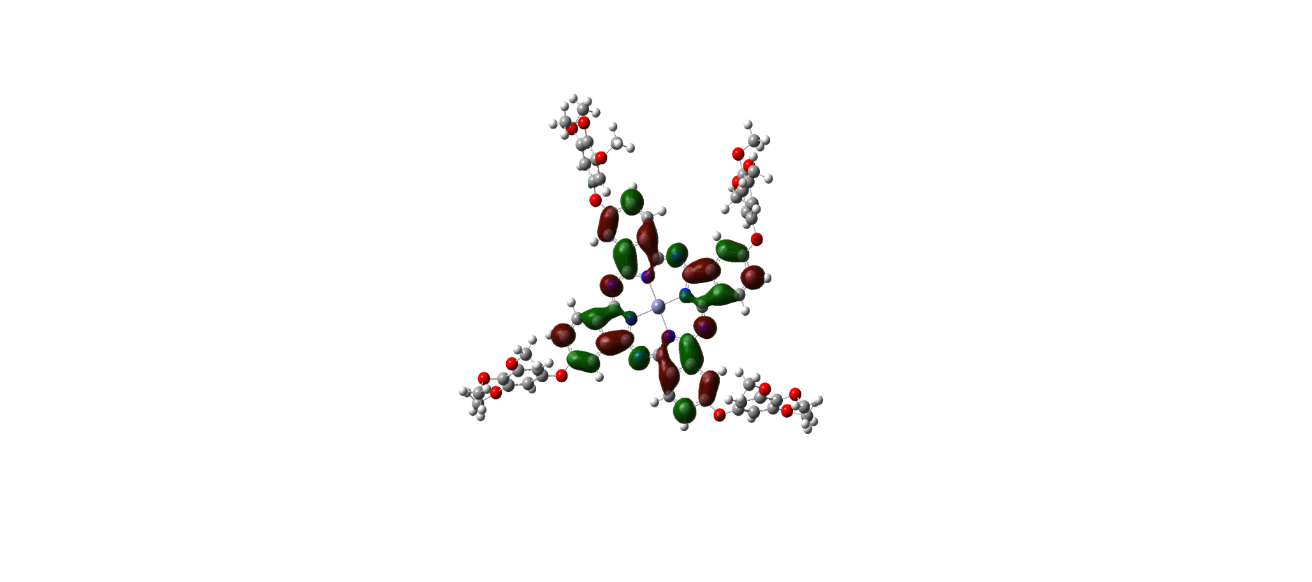


LUMO = -2.60 eV LUMO+1 = -2.59 eV LUMO+2 = 0.94 eV


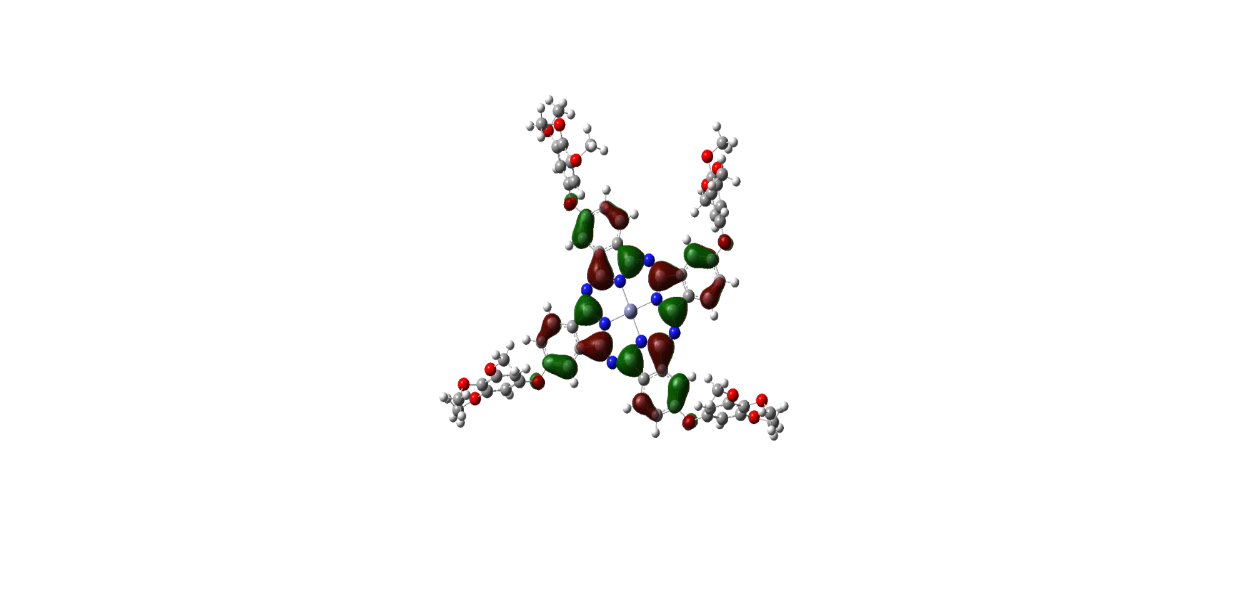

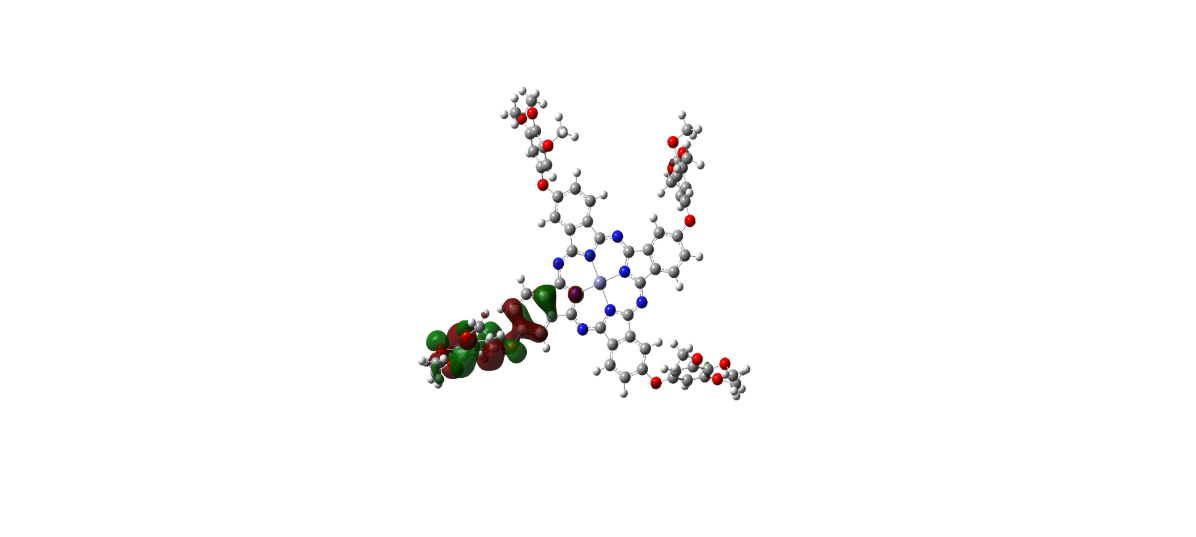

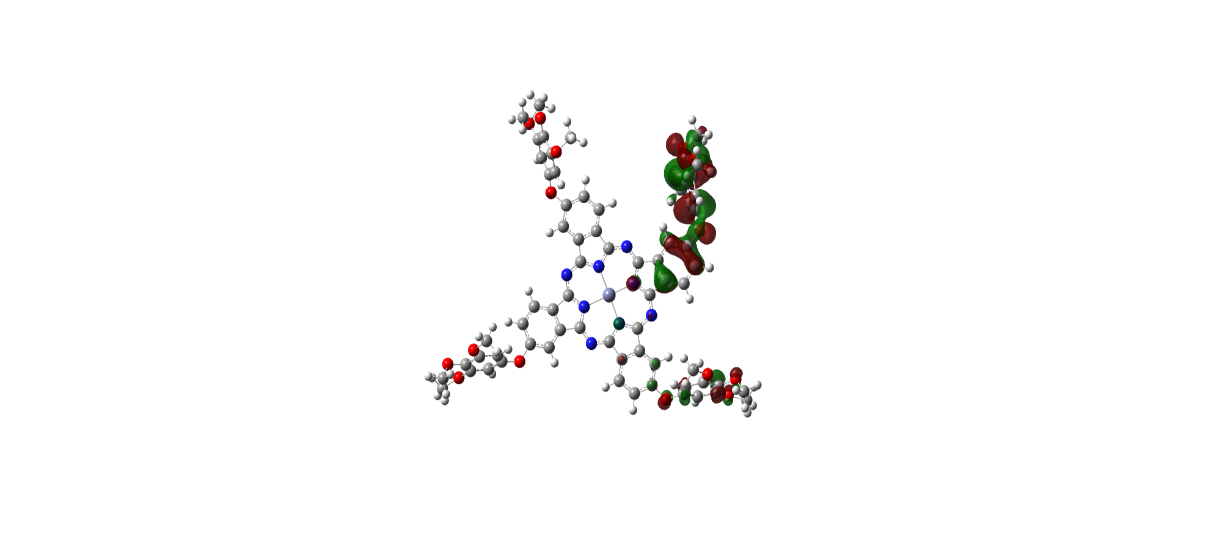


HOMO = -4.74 eV HOMO-1 = -5.65 eV HOMO-2 = -5.69 eV

**Figure S17.**Isodensity plots of FMOs and the energy values in eV by using the B3LYP method 6-31G (d,p) for **ZnTmPc**.

XP-Plus Stylus Profilometer, AMBIOS instrument was utilized to estimate the thickness of the coatings on the three phthalocyanine thin films, made use of in Nonlinear optical study Z-scan experiment. Scanning speed was kept constant 0.03 mm/sec for all the samples. An average thickness of ~ (47-56) µm was estimated through this characterization technique.

**Figure S18.** Surface coating thickness data of the Phthalocyanine thin films (a), (b), (c) for TMPC, Cu-TMPC, Zn-TMPC respectively.


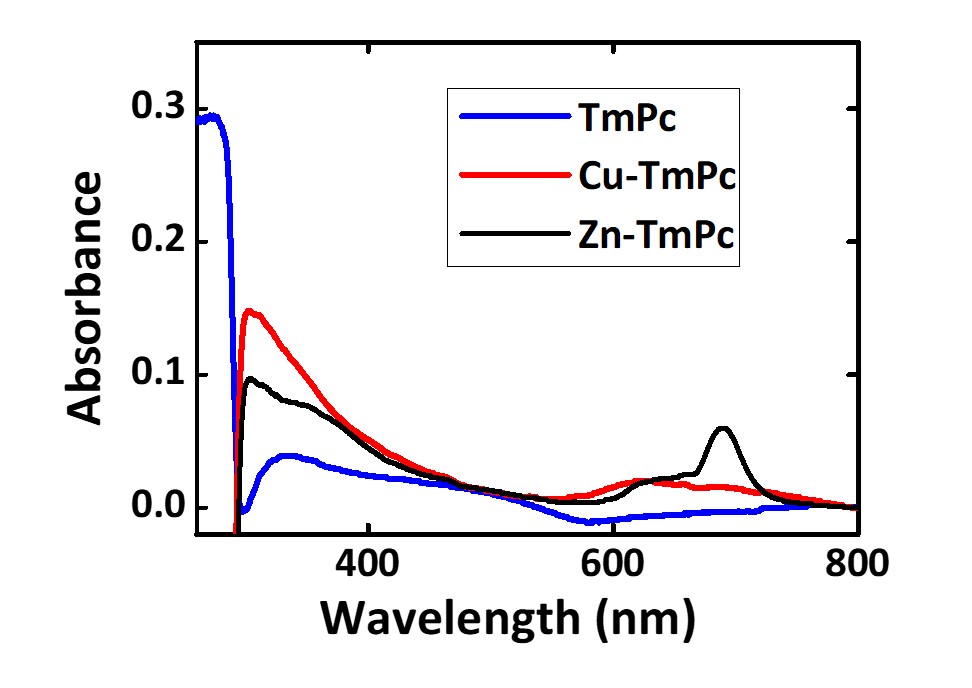


**Figure S19** Absorption spectra of the thin films of phthalocyanines studied in this work.


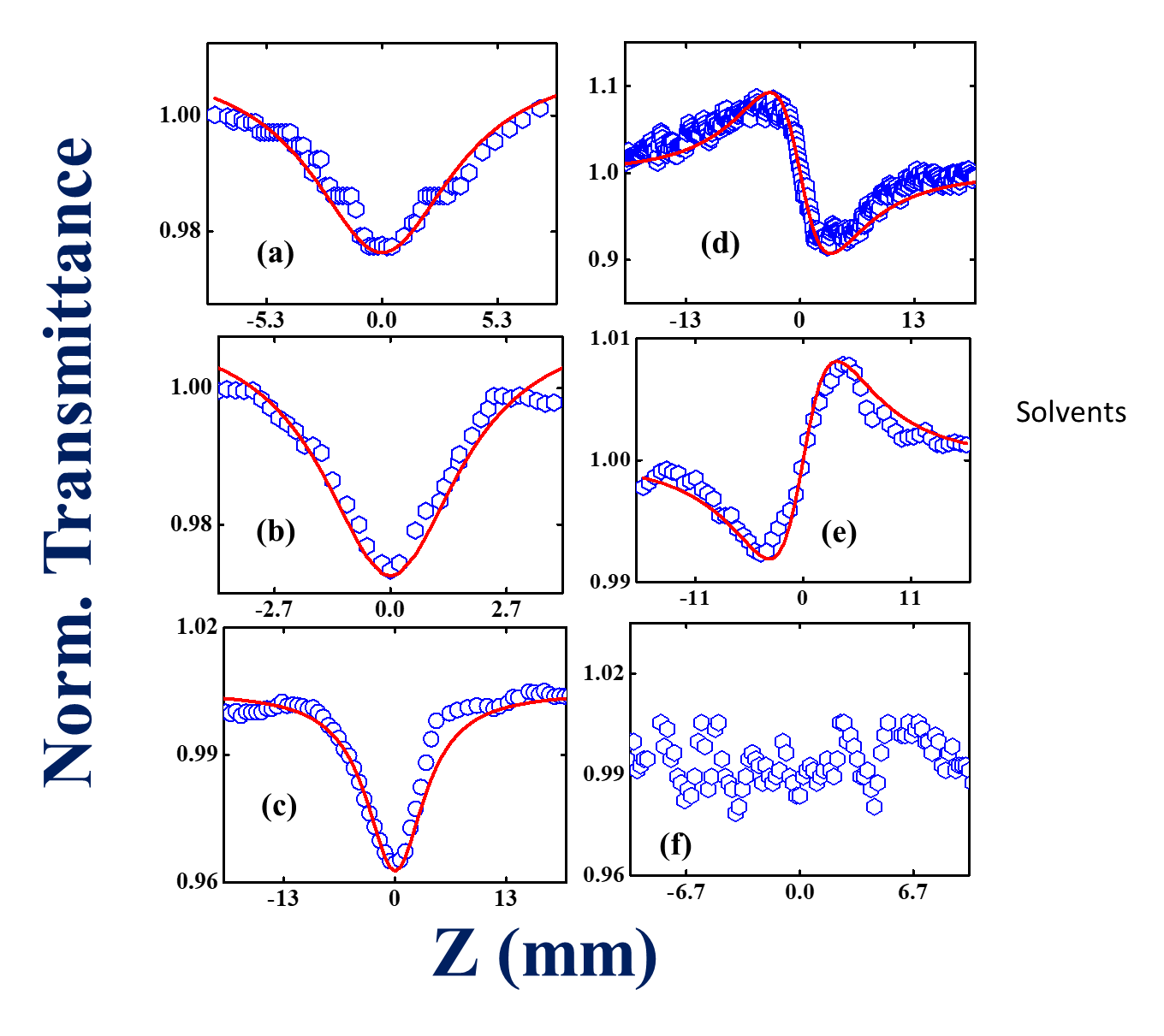


**Figure S20.**Experimental and theoretically fitted Z-scan data of open aperture (a, b) and closed aperture (d, e) for pure dichloromethane (DCM) solvent involving fs MHz, kHz pulses respectively. (c, f) present open and closed aperture data for a clean glass slide in interaction with MHz fs pulses. Open symbols are the experimental data while the solid lines represent theoretical fits.

**Table S1.** Singlet excited state properties of dyes by B3LYP method and M06-2X function in tetrahydrofuran solvent in PCM model.

| **Dye** | **^a^ λ _max_** | **^b^ f** | **^c^ E (eV)** | **% of Molecular Orbital Contribution** |
| --- | --- | --- | --- | --- |
| **TmPc** | 658  341 | 0.7144  0.1345 | 1.88  3.62 | HOMO->LUMO (33%), HOMO->L+1 (60%)  H-8->L+1 (11%), H-1->L+1 (51%) H-13->L+1 (2%), H-12->L+1 (3%), H-11->L+1 (9%), H-9->L+1 (3%), H-3->L+1 (3%), H-1->LUMO (4%) |
| **Zn TmPc** | 645  346 | 0.755  0.1081 | 1.92  3.57 | HOMO->LUMO (95%)  H-11->L+1 (14%), H-8->L+1 (18%), H-2->L+1 (18%), H-1->L+1 (24%) H-14->L+1 (5%), H-12->L+1 (2%), H-10->L+1 (2%) |
| **Cu TmPc** | 644  436 | 0.7303  0.0 | 1.92  2.83 | HOMO(A)->LUMO(A) (46%), HOMO(B)->LUMO(B) (49%)  3.464-A H-9(A)->L+1(A) (5%), H-8(A)->LUMO(A) (8%), H-3(A)->LUMO(A) (4%), H-1(A)->L+1(A) (2%), HOMO(A)->L+3(A) (6%), H-9(B)->LUMO(B) (2%), H-9(B)->L+1(B) (5%), H-8(B)->LUMO(B) (8%), H-3(B)->LUMO(B) (5%), H-1(B)->L+1(B) (2%), HOMO(B)->L+4(B) (5%) |

^a^ Theoretical absorbance in nm, ^b^Oscillator strength, and ^c^Excited state energy in eV.
